# A humanised thrombus-on-a-chip model utilising tissue-engineered arterial constructs: A method to reduce and replace mice used in thrombosis and haemostasis research.

**DOI:** 10.12688/f1000research.158910.1

**Published:** 2025-01-20

**Authors:** Jacob Ranjbar, Jonathan M. Gibbins, Jordan Roe, Paul Roach, Ying Yang, Alan G.S. Harper

**Affiliations:** 1 School of Medicine, Keele University, Keele, England, ST5 5BG, UK; 2 Institute for Cardiovascular & Metabolic Research, University of Reading School of Biological Sciences, Reading, England, RG6 6EX, UK; 3 Department of Chemistry, School of Science, Loughborough University, Loughborough, England, LE11 3TU, UK; 4 School of Life Sciences, Keele University, Keele, England, ST5 5BG, UK

**Keywords:** Tissue Engineering, layer by layer fabrication, thrombosis models, 3Rs, Collagen, Tissue Factor, Mechanical injury model, Microfluidics, thrombus-on-a-chip

## Abstract

The study of *in vivo* thrombus formation has principally been performed using intravital microscopy in mice and other species. These have allowed us to visualise the molecular and cellular processes that regulate thrombus formation inside the body. However current *in vivo* arterial thrombosis models are difficult to standardise between labs and frequently produce results that do not reliably translate successfully in human clinical trials. Here we provide a step-by-step description with accompanying video tutorials to demonstrate how to produce a 3D humanised thrombus-on-a-chip model, which uses perfusion of fluorescently-labelled human blood over a mechanically-injured human tissue engineered arterial construct (TEAC) within a 3D printed microfluidic flow chamber to replicate thrombus formation within a healthy artery. We also provide a written methodology on how to use 3D printing to produce a mechanical injury press that can reproducibly damage the TEAC as a stimulus for thrombus formation as part of a mechanical injury model. Perfusion of the uninjured TEAC with whole human blood containing DiOC6-labelled platelets without initiating notable thrombus formation. The mechanical injury press was shown to induce a reproducible puncture wound in the TEAC. Fluorescence microscopy was used to demonstrate that thrombus formation could be observed reproducibly around sites of injury. This humanised thrombosis-on-a-chip model can replace the use of animals in *in vivo* thrombosis models for preclinical assessment of anti-thrombotic therapies. This method also offers multiple scientific advantages: allowing new drugs to be directly tested on human blood from a diverse array of donors, facilitating use of a realistic and reproducible injury modality as well as removing the potential confounding effects of general anaesthetics in animal studies. The use of human thrombus-on-a-chip models combining TEACs offers a new methodology to reduce animal use whilst improving the predictive capabilities of preclinical trials of anti-thrombotic therapies.

Research highlights **Scientific benefits(s):**

• Standardised means to reproducibly initiate thrombus formation under physiologically-relevant flow conditions in the presence of a 3D construct of the arterial wall
• A humanised-system to better conduct drug development due to interspecies differences in the haemodynamics and molecular biology of platelet receptors
• Collection of blood samples leaving the microfluidic chamber allows post-flow analysis of blood leaving the site of injury, enabling assessment of changes in circulating blood as well as thrombosis and embolus dynamics
• The arterial construct can be further developed to provide thrombosis models to study pathological blood clotting

**3Rs benefits(s):**

• Replace intravital imaging studies performed in mice for pre-clinical drug, nanomedicine and theranostic testing. These type of tests account for 81% of current animal usage in published journal articles
• Adaption of the microfluidic chamber could facilitate use of this model in biocompatibility testing of cardiovascular stents and implants
• The construct could also be used within organ-on-a-chip or body-on-a-chip models to assess thrombotic or bleeding events in other human diseases

**Practical benefits(s):**

• Low cost to produce relative to animal husbandry costs
• Provision of video guides on how to perform the experiments provide practical support to groups looking
• Computer aided design of the microfluidics and mechanical injury press ensures key equipment can be easily reproduced by other groups using a 3D printer. Experiments could be run without imaging equipment through assessment of post-flow blood samples or time to cessation of blood flow.
• Requires relatively small volumes of blood to run an experiment (5 mls)
• Human blood is readily available (with relevant ethical approvals, HTA licenses and/or MTAs in place)

**Current applications:**

• Study impact of anaesthesia on arterial thrombosis
• Studying endothelial progenitor cell homing to sites of vascular damage
• Assessment of current clinically-used anti-thrombotic therapies
• Development of tissue engineered models of early atherosclerosis

**Potential applications**

• High throughput screening of novel anti platelet therapies
• Use in assessing optimal doses for precision medicine approaches to anti-thrombotic therapies or haemostatic agents
• Assessing sex differences in clotting response
• Model of pathological bleeding and thrombotic conditions
• Current efforts to produce human platelets with specific proteins depleted could be used with this model system to produce a humanised system to replace the use of genetically modified mice in thrombosis research

## 1. Introduction

**Figure 1.**
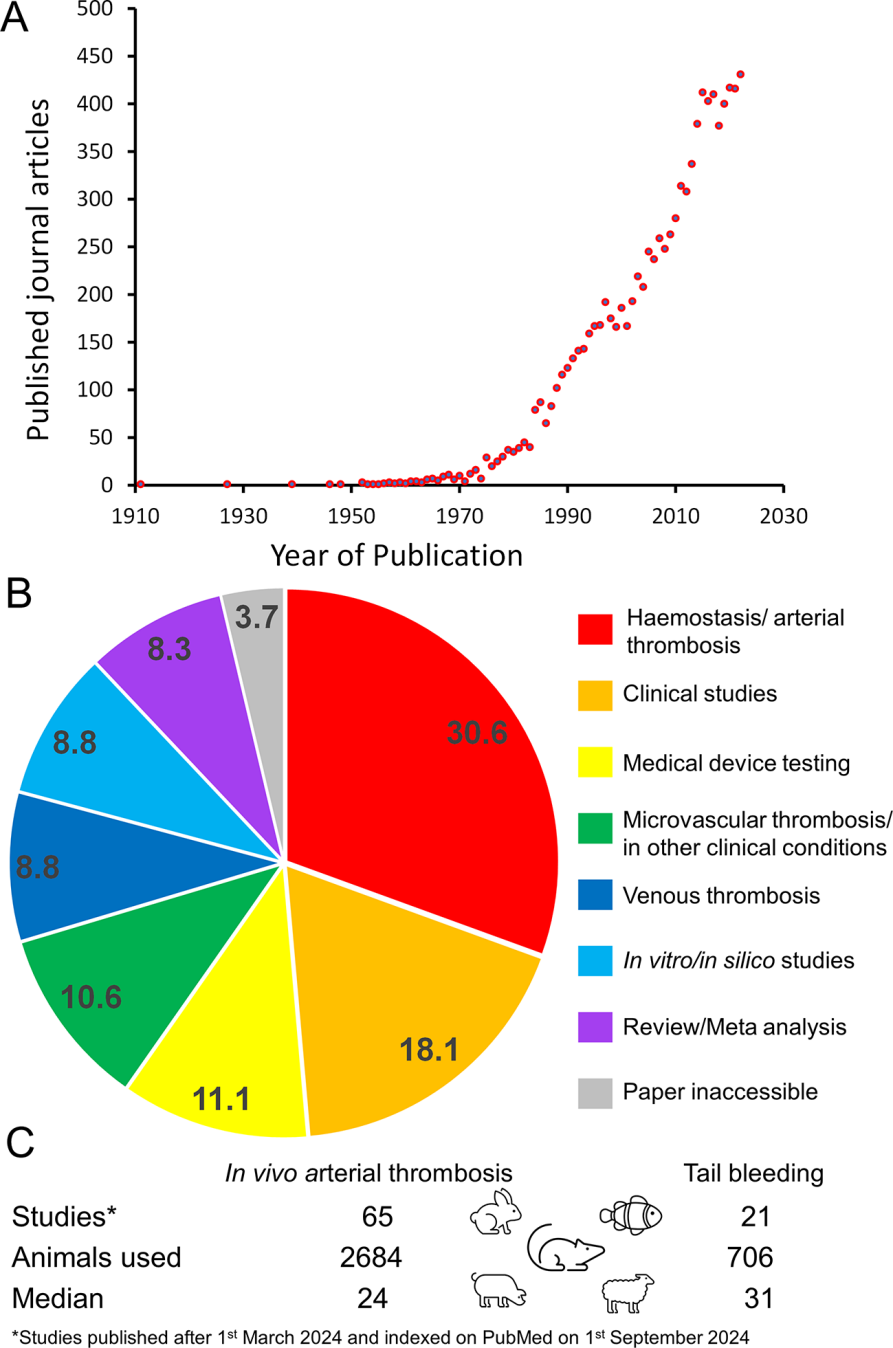
Literature review of current use of *in vivo* thrombosis in models in thrombosis and haemostasis research. (A) A PubMed search was performed for journal articles containing the key phrases “ *in vivo* thrombus” or “ *in vivo* thrombosis” in the abstract. These are presented as numbers of publications in every year since 1910. (B) Classification of the types of in vivo studies performed. Numbers in each segment indicate % of the total studies represented in each category. (C) A summary of the number of studies animals used and the median number of animals in these journal articles published between March-September 2024.

Upon damage to blood vessels, the activation of platelets and the coagulation cascades coordinates the clotting of the blood at the site of injury to prevent excessive blood loss. These processes are regulated by an array of physical and chemical interactions between the damaged arterial wall, plasma proteins, platelets, endothelial cells and leukocytes, plasma as well as blood flow conditions. ^1– 4^ This complex network of stimuli acts to ensure blood clotting occurs specifically at the site of injury and does not occur in undamaged blood vessels. ^3^ However, when these regulatory mechanisms fail, clotting occurs inappropriately inside undamaged blood vessels and can block the blood flow to the heart and brain leading to life- threatening heart attacks and strokes. Acute cardiovascular events are amongst the leading global causes of premature death. ^5^ Because of this there has been increasing interest in studying the processes of thrombus formation *in vivo* ( Figure 1).

The use of intravital microscopy has significantly advanced our understanding of thrombus formation. Intravital microscopy is a non-recovery procedure which involves surgical exposure of accessible arteries and arterioles under anaesthesia in mice, induction of vascular damage and real-time assessment of *in vivo* thrombus formation using fluorescence microscopy. This methodology when combined with drug treatments and transgenic mice, has allowed us to better understand the process of blood clotting at a molecular level. Intravital microscopy is now used widely, with a survey in 2011 noting that 78 groups in at least 11 different countries across 4 continents had published research using this methodology. ^6^ This is likely to be an underestimate of current usage of this technique as the number of journal articles in this area has continued to rise steadily from the 208 articles published in 2004 to the 392 articles published in 2022 ( Figure 1A). Assessment of recent articles found using these search terms that were published between March-September 2024 identified that studies using arterial thrombosis or haemostasis models are the most prevalent (31% of all published papers; Figure 1B). There are also significant number of in vivo studies on other bleeding or thrombotic conditions (19%) or assessment of the biocompatibility of medical devices, such as nanoparticles, vascular stents and artificial heart valves (11%). In contrast, alternative approaches that don’t use animals are in the minority, and including clinical studies using patients (18%), and those recreating in vivo conditions in *in vitro* or *in silico* studies (9%) or performing literature reviews or meta analyses of these studies (8% of studies). Therefore there is significant scope to reduce and replace animal usage in thrombosis and haemostasis research. Assessing animal usage solely in the arterial thrombosis and haemostasis studies found that these used 2684 animals in arterial thrombosis models with a further 706 rodents used in tail bleeding assays over a 6-month period ( Figure 1C). 81% of these studies focused on the effect of administration of drugs, nutrients or nanomedicines on thrombotic responses, with the remaining 19% of animals used in assessing thrombus formation in wild type animals, genetically modified mice, or animals in which complex multi-system changes were induced (e.g. sepsis, burns, cancer). These studies principally used mice and rats, but also utilised pigs, rabbits and zebrafish. From these data we would estimate that around 6780 animals are used in arterial thrombosis and haemostasis research each year, of which 5491 animals would be used for preclinical testing of drugs and other therapeutics.

Despite its increasing popularity previous research has demonstrated that the experimental findings of these arterial thrombosis models are difficult to standardise ^7^ – with results significantly affected by a range of factors including the strain and age of mice used, ^8^ the anatomical location of the injured vessel, ^9^ the type and extent of injury-induced, ^10, 11^ the choice of anaesthesia and ventilation used, ^12^ and the skill of the researcher performing this experiment. ^12, 13^ The results of these studies thus depend at least in part on the experimental set-up as much as the biology and has led to the suggestion that to ensure validity of results, a range of *in vivo* thrombosis models should be used. ^14– 17^ This is consistent with information provided in a review of *in vivo* arterial thrombosis models used in FDA approvals of new anti-thrombotic therapies between 1997 and 2016. ^18^ These submissions used on average (mean) 5 different injury models across 2 species in each drug approval application, with 50% of these also including the use of at least one model in non-human primates. These observations indicate that current *in vivo* arterial thrombosis models are not considered by researchers and regulatory bodies to offer sufficient validity and interlab reproducibility to be considered in isolation for use in discovery research or for regulatory approvals. Tellingly a previous review of cardiovascular therapies tested in animal models indicated that only 21% of those found to be effective in *in vivo* pre-clinical tests elicited patient benefit in subsequent clinical trials. ^19^ This includes every drug developed for the treatment of ischemic stroke, totalling over 1000 failed trials. ^20^ The poor translation into improved patient care indicates that further research is needed to develop improved experimental models to allow us to recreate human thrombosis that reduce and replace the use of animal studies and help bridge the gap between successful pre-clinical studies and clinical trials. In support of this, recently identified anti-thrombotics, such as vorapaxar and glenzocimab, specifically target proteins only found on human (and not mouse) platelets. ^21^

One proposed solution is the development of humanised thrombus-on-a-chip models. ^22– 25^ These models principally use microfluidic flow chambers coated with endothelial cells, equine fibrillar collagen (Horm) and/or recombinant, lipidated human tissue factor to replicate the intimal lining and the subendothelial matrix. ^25, 26^ Thrombus formation is then monitored using fluorescence imaging of platelets when perfused with freshly-donated human blood samples under physiological blood flow conditions. These provide a cheap and accessible microphysiological system in which to replicate the basic initiating steps of haemostasis, providing a viable alternative to current *in vivo* studies. These systems would be well suited to the replacement of animals used in preclinical testing of new therapies aiming to combat thrombotic conditions or bleeding disorders. Whilst these *in vitro* thrombosis models offer the possibility of monitoring human thrombus formation in real-time, there are several potential issues with current models. Firstly, flow chambers generally use coated PDMS and glass surfaces to form the flow channel, which have significantly greater mechanical stiffness than the arterial wall (30-40 kPa vs > 1GPa). ^27^ As substrate stiffness is known to influence both platelet activation and endothelial cell permeability, ^28^ these mechanical properties may impact on the validity of the simulation. Additionally, the use of equine fibrillar collagen could overstate the clotting responses according to a previous study that compared the thrombotic responses seen in a standard microfluidic flow chamber coated with Horm collagen, Human Fibrillar type I or Human fibrillar type III collagen when coated on the same microfluidic chamber. ^29^ These studies demonstrated that coating microfluidic chambers with Horm collagen resulted in greater surface area coverage by the thrombi than seen with the human fibrillar collagens. This was associated with a differential impact on the expression of phosphatidylserine (a procoagulant acidic phospholipid) on the surface of thrombi. Marginal phosphatidylserine exposure was observed in thrombi produced on human fibrillar collagen coated surfaced whilst strong expression was observed on Horm collagen exposed blood samples. These results that use exogenous collagen sources may overstate the thrombotic response and could alter their sensitivity to drug treatment. Previous reports have identified the difficulty in standardising collagen coating of microfluidic chambers to provide a uniform pro-thrombotic environment, ^30^ especially using commercial fibrillar collagen sources that have been shown to have batch-to-batch variation. ^31^ Similarly recombinant tissue factor has shown to possess reduced glycosylation relative to affinity purified placental tissue factor produced in E Coli or Sf9 insect cells, and that this correlates with a significantly reduced activation of Factor X when incubated with Factor VIIa. ^32^ The use of exogenous thrombogenic substrates coated on artificial surfaces may alter the way thrombi form and respond to potential treatments thus impacting on their ability to offer improved translation of findings from bench to bedside.

Previously our lab has developed a humanised *in vitro* arterial thrombosis model, produced through using vascular tissue-engineering techniques to produce a 3D human arterial construct (TEAC). The intimal layer is created by culturing human umbilical vein endothelial cells (HUVECs) on top of fibronectin-coated aligned polylactic acid (PLA) nanofibers. Simultaneously, a medial layer construct was grown by culturing human coronary artery smooth muscle cells (HCASMCs) within a 3D type I collagen hydrogel. ^33, 34^ These arterial constructs provide a 3D structure that better replicates the structure and substrate stiffness of the native vessel wall (34 kPa). A previous study found that collagen hydrogels have stiffness’ ranging from 6-15kPa. ^35^ Our previous studies have demonstrated that when intact, this human arterial construct is able to prevent thrombus formation and inhibits platelet activation both under static and physiological flow conditions. In contrast, when the medial layer is exposed without an intimal lining, or the intimal lining of the arterial construct is damaged this construct can support thrombus formation under physiological flow conditions. ^33, 34^ The medial layer biomimetic hydrogel acts to trigger thrombus formation through endogenous production of tissue factor and collagen by the HCASMCs, that can elicit activation of both the primary and secondary haemostatic pathways upon exposure to the bloodstream. ^7, 33^ Through eliciting production of endogenous human activators of thrombus formation, the human arterial construct provides a thrombogenic substrate that has the potential to accurately model thrombus formation. This construct therefore provides a direct alternative for recreating commonly used animal and thrombosis models to reduce and replace animals used in preclinical research.

In this article, we provide the protocol for producing a humanised thrombus-on-a-chip model by designing a method to encapsulate a modified version of the TEAC in a 3D printed microfluidic chamber. This TEAC is able to resist thrombus formation when uninjured, but support reliable thrombus formation under physiological flow conditions after injury with a mechanical injury press. This provides a methodology to recreate human thrombus formation under near-physiological conditions that could be used as a viable alternative to current arterial thrombosis models used to study *in vivo* thrombus formation in animals.

To facilitate the uptake of this method, we have produced a series of video tutorials to provide a visual guide to support other groups in adopting this technique.These are referenced within the protocol section to indicate which video each section can be found in and a playlist is provided at the end of this article to give specific timings for each technique. The videos can be downloaded from the Keele University data repository alongside 3D printing files required to construct the *in vitro* thrombosis model. ^36^

## 2. Methods

### 2.1 Materials

2.1.1 Materials for Tissue Engineered Arterial Construct

**Table:** 

Product	Company	Notes
Human Coronary Artery Smooth Muscle cells (HCASMCs)	Fisher Scientific (Loughborough, UK)	Cells for Tissue Engineered Medial Layer Primary human cells must be stored, used and disposed of in premises with a human tissue authority license Used up to Passage 5
Human umbilical vein endothelial cells (HUVECs)	Fisher Scientific (Loughborough, UK)	Cells for Tissue Engineered Intimal Layer Primary human cells must be stored, used and disposed of in premises with a human tissue authority license Used up to Passage 5
Human Vascular Smooth Muscle Cell Medium (previously known as Medium 231)	Fisher Scientific (Loughborough, UK)	Media for HCASMCs culture Must be kept sterile
Human Large Vessel Endothelial Cell Basal Medium (previously known as Medium 200)	Fisher Scientific (Loughborough, UK)	Media for HUVEC culture Must be kept sterile
Smooth Muscle Growth Supplement (SMGS)	Fisher Scientific (Loughborough, UK)	Media for HCASMC culture Must be kept sterile
Low Serum Growth Supplement	Fisher Scientific (Loughborough, UK)	Growth Supplement for HUVEC culture Must be kept sterile
α-MEM medium powder	Fisher Scientific (Loughborough, UK)	Component of medial layer hydrogel Used to produce a sterile 10x stock solution
CellTrace ^TM^ Violet Proliferation Kit	Fisher Scientific (Loughborough, UK)	Fluorescent cell label used to track culture of HUVEC growth on nanofibers Must be kept sterile
Corning High Concentration Rat Tail Type I Collagen	Fisher Scientific (Loughborough, UK)	Component of medial layer hydrogel Must be kept sterile
Whatman Qualitative Filter paper	Scientific Laboratory Supplies (Nottingham, UK).	Used to produce filter paper frames Autoclave to sterilise prior to use
Lonza Penicillin-Streptomycin-Amphotericin B	Scientific Laboratory Supplies (Nottingham, UK).	Prevents infection of primary cell cultures Must be kept sterile
L-Ascorbic Acid	Sigma Aldrich (Gillingham, UK)	Supports enhanced thrombogenicity of medial layer by supporting HCASMC collagen production and tissue factor activity Must be kept sterile
DETA-NONOate	Cambridge Bioscience (Cambridge, UK).	Nitric Oxide donor to inhibit medial layer gel contraction Must be kept sterile
Human Fibronectin Protein, CF	R&D Systems (Abingdon, UK)	PLA nanofibre coating to improve HUVEC adherence and proliferation Must be kept sterile

2.1.2 Materials for the microfluidic chamber

**Table:** 

Product	Company	Notes
Elegoo Mars 2 Pro MSLA Printer	Amazon UK	Printing of the microfluidic flow chamber
Elegoo Mercury Wash & Cure Station	Amazon UK	Production of the microfluidic flow chamber
Elegoo Standard Translucent Resin	Amazon UK	Resin used to produce the microfluidic flow chamber
8 mm Linear Bearings	Amazon UK	Component of the mechanical injury press
Linear Rods (8mm x 15cm)	Amazon UK	Component of the mechanical injury press
Precision dispenser needles 18-gauge	Scientific Laboratory Supplies (Nottingham, UK).	Used as a flow adaptor for the microfluidic flow chamber
Haemocytometer coverslip	Scientific Laboratory Supplies (Nottingham, UK).	Used to provide an optically transparent window that compresses the tissue engineered arterial construct onto the
Terumo Surflo Winged Butterfly Infusion Set 19G Ivory	MediSupplies (Poole, UK)	Used to connect syringe pump to the microfluidic flow chamber
M3 Threaded Inserts	RS Components (Corby, UK)	Used to connect the compression chip to the microfluidic flow chamber
M3 Bolts 10mm	RS Components (Corby, UK)	Used to connect the compression chip to the microfluidic flow chamber
PETG Filament	RS Components (Corby, UK)	Used to produce the PETG frame that holds the nanofibers for the TEIL culture
Polylactic acid (PLA) filament	RS Components (Corby, UK)	Used to 3D print components of the mechanical injury press
26-gauge needles	RS Components (Corby, UK)	Needle head used for the mechanical injury press
Compressed air	RS Components (Corby, UK)	Used to remove debris from microfluidic chip
Fluid Transfer Tygon S3 E-3603 Transparent Process Tubing, 0.8mm	RS Components (Corby, UK)	Used to perfuse microfluidic chip
Ball bearings (10x15x4mm)	RS Components (Corby, UK)	Used in the mechanical injury press to ensure smooth lowering and raising of the needle head.

### 2.2 Methods

2.2.1 Assessment of the reproducibility of the mechanical injury press

TEACs were prepared following the protocol outlined in Section 2.3.2 Initially, the culture medium was aspirated from the constructs, which were then washed twice with HEPES-buffered saline (HBS) for 5 minutes each. Following the initial wash, the constructs were carefully transferred face down onto a polytetrafluoroethylene (PTFE) plate using tweezers, taking care to avoid contact with the interior of the frames. The constructs on the PTFE plate were then subjected to mechanical injury using a specialised press. This was achieved by lowering a needle head to ensure the needle tips penetrated through the entire gel thickness. The needle head was equipped with three evenly spaced 27-gauge needles to puncture the centre of the top and bottom lane of the gel constructs (one hole per lane) of three separate TEACs. Subsequently, the injured gel constructs were transferred back into a 12-well plate, positioned face up, and washed with HBS. The washing procedure involved a single 5-minute wash followed by two 10-minute washes under agitation. After washing, the samples were stained using the Live/Dead Cell Staining Kit II to distinguish between viable and non-viable cells. ImageJ software (1.53K; https://imagej.net/ij/) was employed to measure the largest diameter of the physical disruption (visible in light microscopy) as well as the area affected by cell damage (indicated by red fluorescence) in each of the channels perfused with human blood (i.e. a measurement was made for both). Measurements of both the physical hole and the size of the zone of cell death were determined by drawing a line across the largest dimension of the respective areas for each hole produced.

2.2.2 Assessment of the reliability of thrombotic response in the flow study

This study received approval from the Keele University Research Ethics Committee (Study reference: MH-200152; Approval date: 1 ^st^ December 2020) and was conducted in accordance with the Declaration of Helsinki. Blood was donated by healthy volunteers under written informed consent. The preparation of whole blood containing CFSE-labelled platelets was performed using the methodology outlined in Section 2.3.4. CFSE-labelled whole blood was perfused for 10 minutes over either intact (no injury) TEACs or TEACs that were mechanical injury in both the top and bottom channel of the chip. The non-injury control experiments were run on separate TEACs produced from the same batch as the MI TEACs. This approach was taken to eliminate any potential influence of the effect of injury of distal channels on the non-injury response. 10 constructs produced from 4 independent cell cultures were used for these experiments.

When assigning groups, TEACs were randomly placed into well plates and allocated to their respective groups on the day of production based on position within the well plate. No formal randomization process was applied to decide which position each TEAC was put into the well plate. Fluorescence imaging was conducted using a Leica inverted fluorescence microscope (MSV269), employing an excitation wavelength of 485 nm and an emission wavelength of 501 nm. Still photos were captured every 30 seconds within the microfluidics channels. Images from the 5-minute time-point, encompassing both mechanical injury and no-injury controls, were analysed to assess the adhesion and aggregation of CFSE-labelled platelets to the tissue-engineered arterial constructs (TEACs) within the microfluidic channels. The quantification of the thrombotic response entailed the use of ImageJ software, assessing the extent of platelet aggregation and thrombus formation. This was achieved through measuring the mean pixel CFSE fluorescence across the entire strip of each individual channel indicating the extent of thrombus formation on the TEAC. No blinding was used during data analysis.

2.2.3 Optical coherence tomography analysis

Optical coherence tomography (OCT) imaging was carried out using a commercial instrument, Thorlabs Telesto II spectral domain OCT system with central source wavelength 1310 nm and 5.5 μM axial resolution in air, enabling cross thickness imaging and 3D reconstruction. Samples for OCT imaging included the entire mechanical injury thrombosis model with the microfluidics bracket, microfluidics chip and mechanically injured TEAC still integrated after a post flow study.

2.2.4 Statistical analysis

Data are presented as Mean ± SEM. A paired Student’s t-test was performed on GraphPad Prism (JASP can be used as an open source alternative; https://jasp-stats.org/) to assess statistical difference in the thrombotic response of the top and bottom channel of the microfluidic chamber of each TEAC. P < 0.05 was considered statistically significant.

### 2.3 Protocols

2.3.1 Producing the microfluidics System (Videos 1 and 2)

A 3D printed microfluidics system is used to house the TEAC and allow for perfusion of freshly-donated whole human blood samples over the construct under physiological flow conditions. The microfluidics system is specially designed for the 3D hydrogel structure and consist of a microfluidics chip and a compression bracket to ensure sealing of the interface between the chip and human arterial construct to prevent blood leakage under flow conditions. As the microfluidic system is used after the completion of the TEAC culture, this does not need to be performed under sterile conditions unless the investigator wishes to track TEAC responses following the completion of the perfusion experiment.

Printing the resin parts (Video 1)

**Figure 2.**
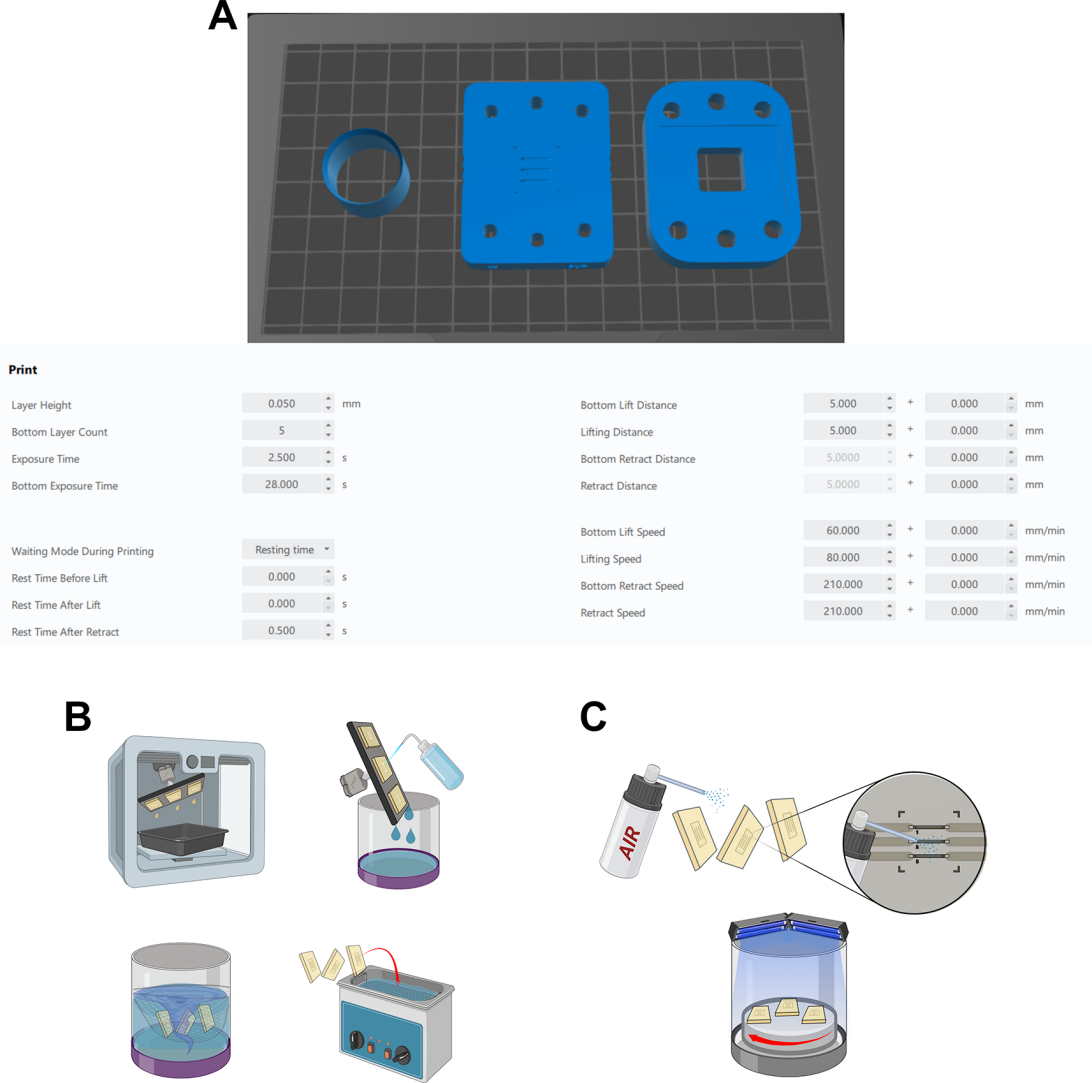
Printing the microfluidic chip. Figure created with BioRender.com. (A) Load .stl models into Chitubox software. Prepare slices for printing using parameters shown. (B) Allow excess resin to drain from the printed chambers and then wash with IPA and sonic bath to remove uncured resin (C) Dry and then cure the printed parts.

1. Download the microfluidics chip, microfluidics compression bracket and TEIL cutter .STL files from the Keele University data repository. ^36^
2. Open the Chitubox Basic slicing software (or other slicing software designed for MSLA printers). Chitubox Basic is freely available and can be downloaded from https://www.chitubox.com/en/index.
3. Load the .STL models into the software using the model orientation shown in Figure 2A.
4. For the microfluidics chip rotate the model so the channels of the model are facing up and the base of the model is flat on the build plate. Ensure channels are parallel to the x-axis of the build plate.
5. For the compression bracket, rotate the model so that the coverslip inset is facing away from the build plate.
6. For the TEIL cutter ensure the flat side of the model is on the build plate with the ‘sharp’ edge orientated upward, away from the build plate.
7. Slice the models using the guide parameters shown in Figure 2A.
8. Save the sliced .ctb file to a USB storage device and transfer to the Elegoo resin printer. 3D printers with equivalent printing resolution could also be utilised after appropriate calibration of the printer settings.
9. Ensure the printer is correctly set up and calibrated using the manufacturers manual.
10. Thoroughly shake the Elegoo transparent resin bottle prior to use to ensure it is thoroughly mixed.
11. Fill the printer vat with the Elegoo transparent resin.
12. Wait for the resin to settle and the bubbles formed during the shaking step to subside (10 minutes).
13. Ensure the build plate is securely in place.
14. Close the lid of the printer and load the pre sliced model file onto the printer and press start.
15. After printing is complete, allow sufficient time for uncured resin to drip into the vat ( Figure 2B).
16. Fill the Elegoo mercury wash cure container with >95% isopropyl alcohol (IPA).
17. Take the build plate off the printer and hold over the container.
18. Using a squeeze bottle, spray >95% isopropyl alcohol onto the resin parts to remove uncured resin from areas with small detail (e.g., microfluidics channels).
19. Place the container into the Elegoo mercury wash & cure machine.
20. Use the printer’s scraper tool to pry the parts off the build plate.
21. Place the parts in the wash basket and into the container with isopropyl alcohol.
22. Wash the parts for 10 minutes using the wash & cure machine.
23. Remove parts and place into ultrasonic bath with >95% isopropyl alcohol.
24. Run the sonic bath on high for 15 minutes to clean any remaining uncured resin from the parts.
25. Remove the parts from the bath and use compressed air to aid in the drying ( Figure 2C).
26. Place the parts in the Elegoo mercury cure machine and allow to cure for 90 seconds (repeat curing cycle if the parts are still ‘tacky’).

Preparation of microfluidics system

This section details how to prepare and attach peripherals to the resin printed parts. Adhering the inlets and outlets to the microfluidics chip as well as how to adhere the coverslip and threaded inserts to the compression bracket are detailed here.

Preparation of barbed inlet and outlet tubes

**Figure 3.**
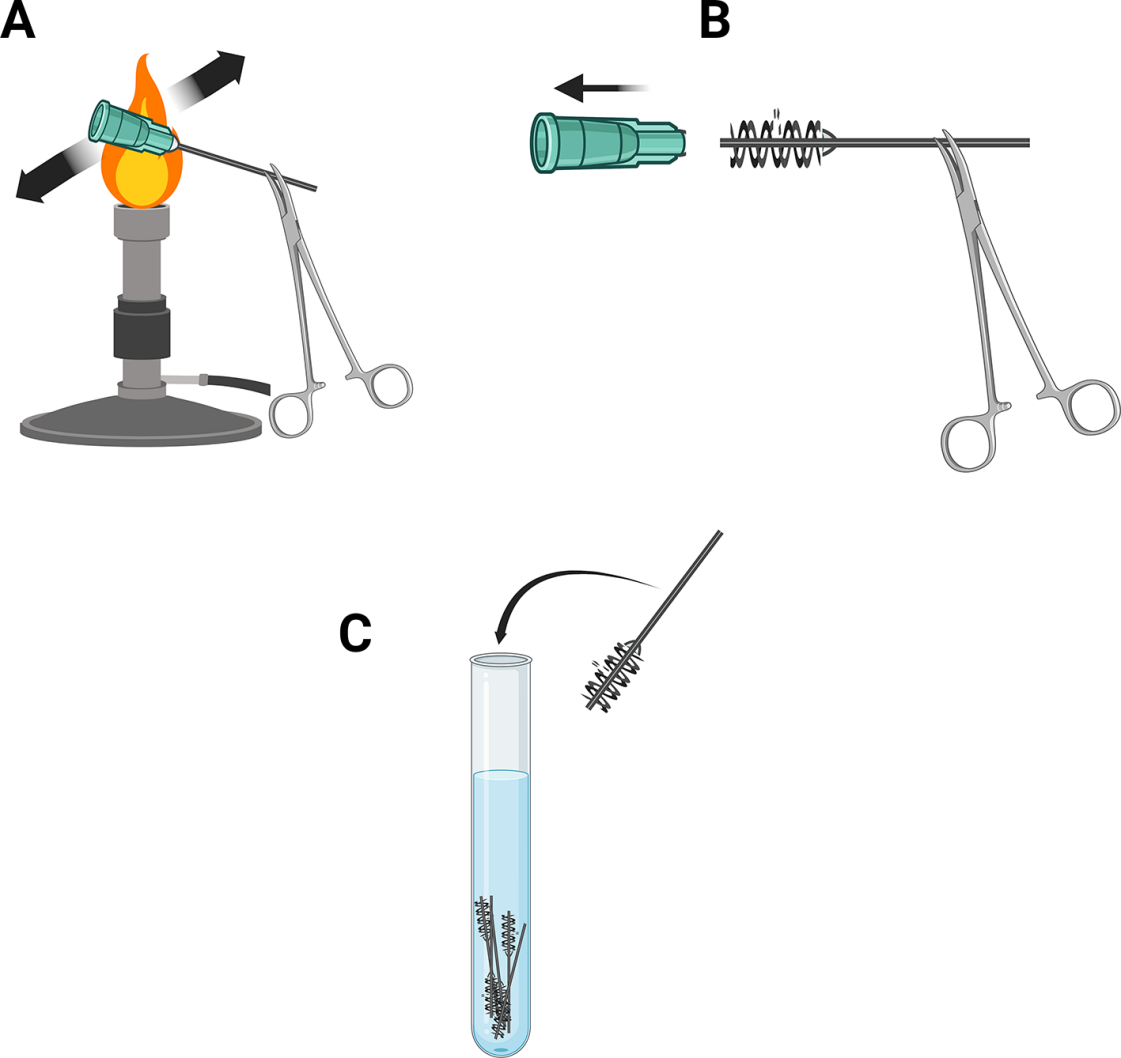
Preparation of barbed inlet and outlet tubes. (A) Pass the plastic end of an 18-gauge needle through a heat source (B) pull plastic housing from the needle (C) Coll needles in distilled water prior to use or storage. Figure created with BioRender.com.

1. Start a Bunsen burner or other appropriate heat source able to heat the plastic enough to allow easy removal of needle.
2. Use pliers to grab 18-gauge dispenser needle by the metal tip.
3. Pass the plastic end rapidly through the flame or heat source 3-4 times ( Figure 3A).
4. Use paper towels to pull the plastic housing off the needle leaving behind the metal tube and rubber barbs ( Figure 3B).
5. Drop the needles into a container filled with distilled water to cool them before handling and storage ( Figure 3C).
6. Dry before use.

Assembly of the microfluidics chip

**Figure 4.**
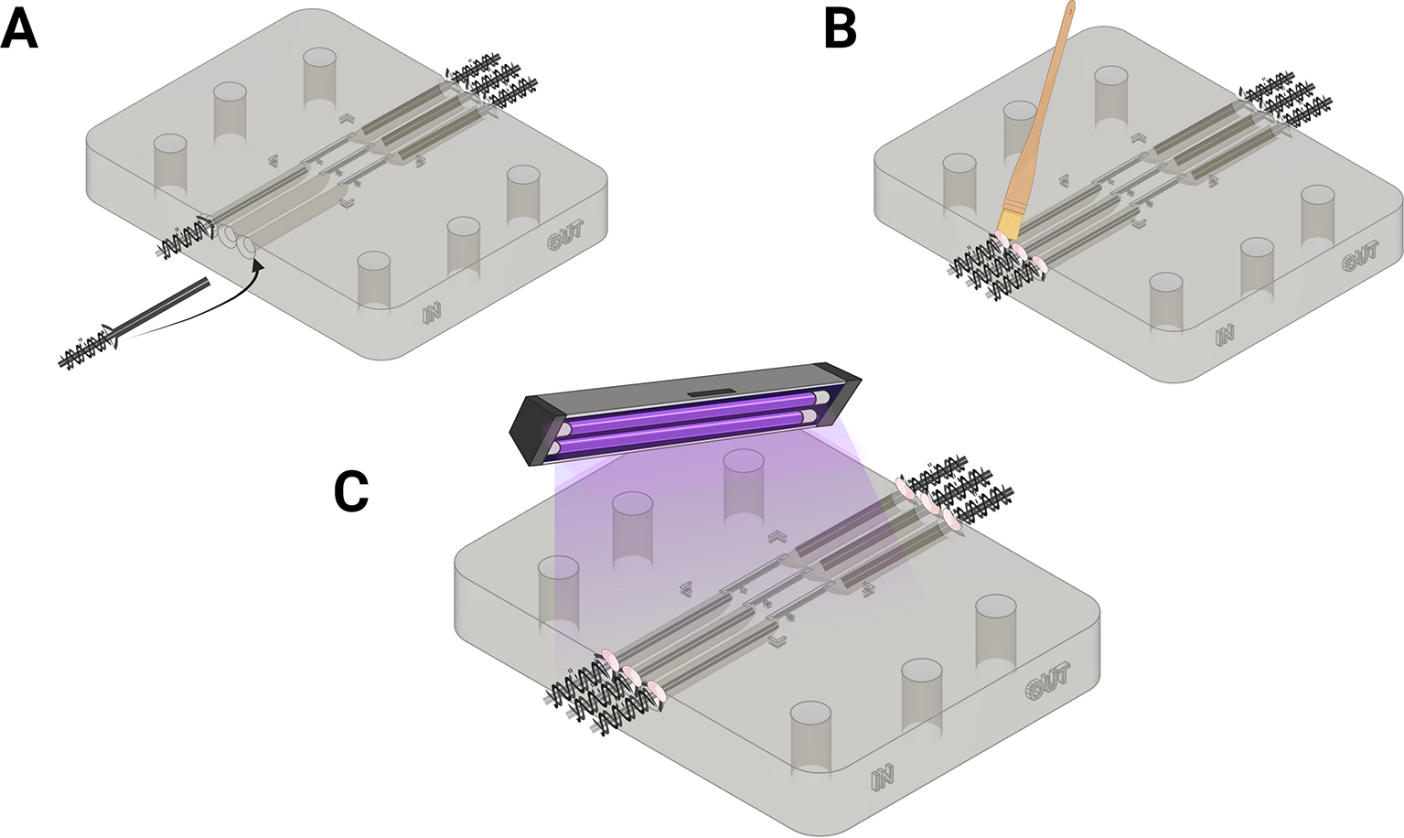
Assembly of the microfluidics chip. (A) Insert the barbed ending of the needles to produce the inlets for the microfluidic chip. (B) Seal the inlets by coating with resin (C) Cure to fix the inlets in place. Figure created with BioRender.com.

1. Insert the barbed metal tubing prepared in the previous section, using the non-barbed end into the inlets of the microfluidics chip ( Figure 4A).
2. Use a small paintbrush to introduce Elegoo transparent resin into the interface between the barbed end of the tube and the microfluidics chip inlet, ensuring that there is no area left unpainted ( Figure 3B).
3. Repeat step 2 until all inlets have been painted with the Elegoo transparent resin.
4. Use a 405 nm light source to cure the resin between the metal tubing and resin chip for 2 mins ( Figure 3C).
5. When fully set, apply a light pulling force to the metal tubing to ensure they have correctly adhered in place.
6. Repeat step 1-5 for the outlets of the microfluidics chip.

Assembly of the compression bracket

**Figure 5.**
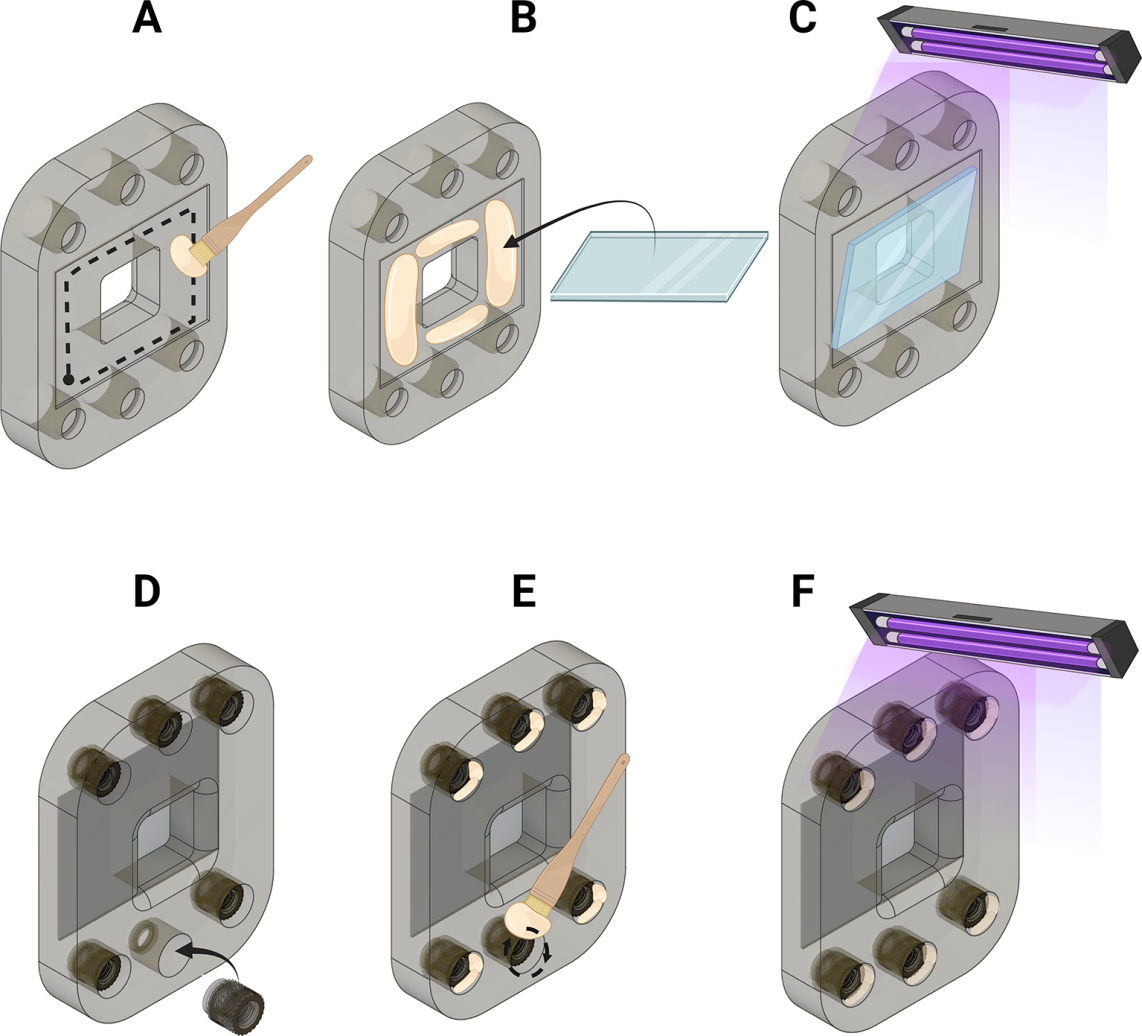
Assembly of the compression bracket. (A) Coat the coverslip inset with resin (B) Attach the coverslip (C) Cure with UV light to affix. (D) Insert threaded insets into the holes of the compression brackets (E) Coat exposed surfaces of the threaded inserts with resin (D) Cure to fix inlets into the compression bracket. Figure created with BioRender.com.

1. Use a paintbrush to lightly paint the inset of the compression bracket with Elegoo transparent resin. Ensure all area is sufficiently covered without any gaps ( Figure 5A).
2. Remove the haemocytometer coverslip from its packaging and place into the painted inset of the compression bracket ( Figure 5B).
3. Using gloved hands, apply even pressure to the coverslip ensuring the coverslip makes good contact with the uncured resin.
4. Place a 405 nm UV light over the coverslip and allow it to cure and adhere to the resin- coated part for 2 minutes ( Figure 5C).
5. Liberally paint the outside of the m3 threaded inserts and place into recessed holes located on the top side of the compression bracket to secure them in place. Ensure no resin enters the threaded area of the inserts ( Figure 5D).
6. Use the paintbrush to apply Elegoo transparent resin into the gap between the threaded insert and the compression bracket ( Figure 5E).
7. Place under 405 nm UV light source for 5 minutes ( Figure 5F).
8. Test whether the resin is cured correctly (non-tacky). If resin is uncured repeat step 7.

Assessing the quality of the microfluidics chip

**Figure 6.**
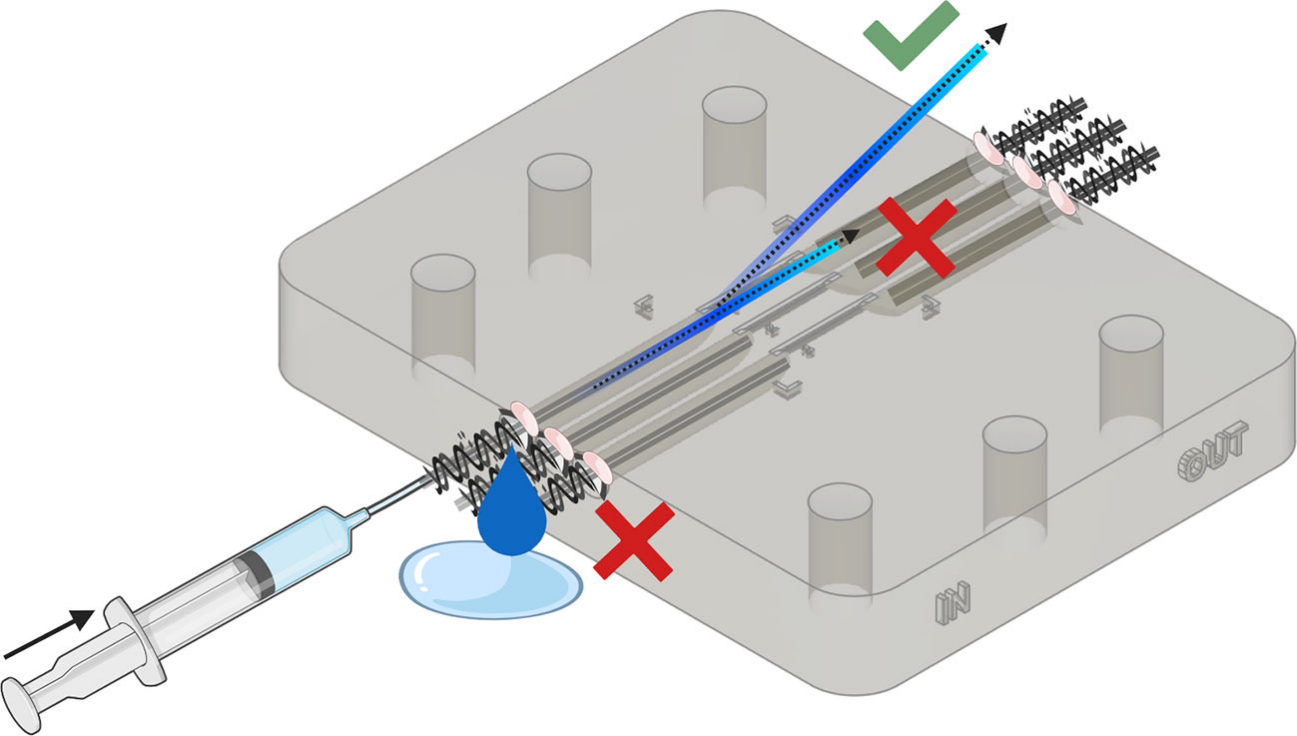
Assessing the quality of the microfluidics chip. Push IPA through each flow channel and ensure that flow is not hindered, continuous, laminar and emerges at the centre of the channel and at a ≈45° angle away from the surface of the channel. Figure created with BioRender.com.

To ensure that the microfluidics chip has been prepared correctly, the channels can be assessed and observed for signs of occlusion or inconsistent flow ( Figure 6).

1. Use a syringe with attached Tygon Tubing to aspirate 6 mL of >95% isopropyl alcohol.
2. Attach the other side of the tubing to the inlet of the microfluidics chip
3. Hold the microfluidic chip over a sink or appropriate container
4. Using reasonable force, push 3 mL of IPA into the microfluidics channel
5. Visually assess the following:
  • Liquid exiting the inlet of the microfluidic channels located at the centre of the chip.
  • No obvious occlusion.
  • Flow of liquid is laminar and continuous.
  • Flow is approximately at a 45-degree angle away from the plane of the channels.
6. Use your thumb to cover the exposed microfluidic channels.
7. Using reasonable force, push the remaining 3 mL of isopropyl alcohol into the inlet of the chip. Assess whether alcohol is seen exiting the adjacent outlet.
8. Repeat step 1-7 for all inlets and outlets of the microfluidics chip.
9. Repeat step 1-8 using PBS or dH _2_O to washout remaining isopropyl alcohol.

2.3.2 Producing the TEAC (Videos 3-5)

The tissue engineered arterial construct (TEAC) comprises a medial layer and intimal layer that are cultured separately and combined prior to experiments. The intimal layer consists of highly aligned poly lactic acid (PLA) nanofibers (Video 3) upon which human umbilical vein endothelial cells (HUVECs) are cultured (Video 4). The medial layer is produced using Rat tail collagen and human coronary artery smooth muscle cells (HCASMCs; Video 5). The layer-by- layer construction strategy for the human arterial construct to allow for independent culture of the TEIL layer and TEML hydrogels to maximise quality of both layers.

TEIL Fabrication

The TEIL is fabricated by seeding human umbilical vein endothelial cells (HUVECs) onto an aligned PLA nanofiber mesh suspended on a 3D printed circular PETG frames using biocompatible UV curable resin as the adhesive.

Production of the nanofiber mesh (Video 3)

Aligned PLA nanofibers are used to guide the attachment and alignment of HUVECs in the TEIL to guide the development of aligned cells seen in the intimal lining of the native blood vessel.

**Figure 7.**
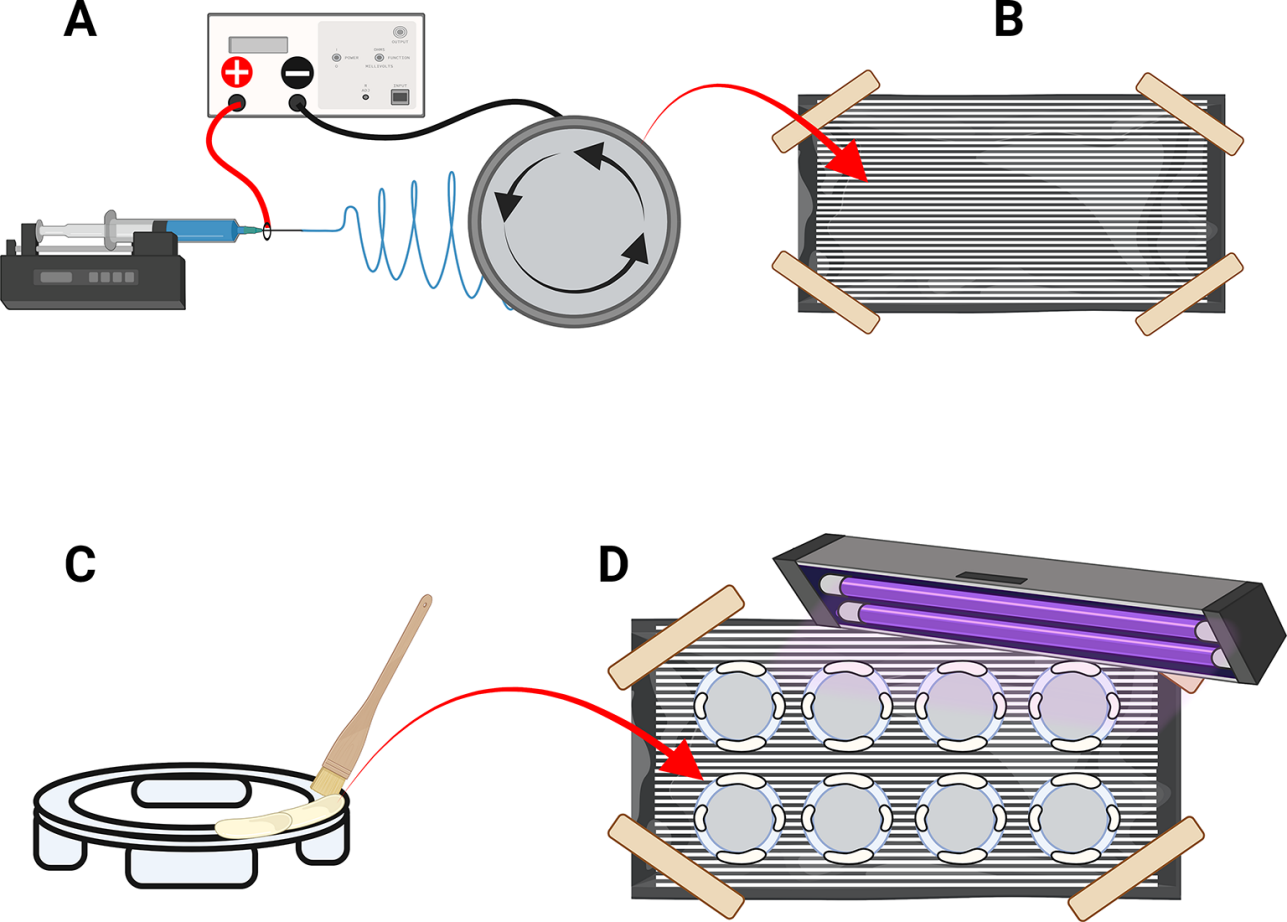
Production of the aligned nanofibre frames for the TEIL. (A) Electrospin aligned PLA nanofibers onto an aluminium fold support on a rotating drum. (B) remove aligned nanofibers from the rotating drum (C) paint on resin to the 3D printed nanofibre frame (D) press down ono the align nanofibers and cure using uv light to secure. (E) remove the aluminium foil from the attached nanofibers (F) transfer to well plate for sterilisation and coating with fibronectin prior to use. Figure created with BioRender.com.

1. Nanofibers are fabricated by electrospinning using a 3% [w/v] PLA polymer solution. For 5 ml polymer solution, dissolve 150 mg PLA polymer granules into 3.5 mL chloroform in a screw top glass container within a fume hood. Place a magnet stirrer into the solution and close the lid.
2. Leave the solution to mix overnight at room temperature under continuous magnetic stirring on a slow spin speed (to prevent bubble formation).
3. Ensure the PLA granules are fully dissolved in solution before moving on to the next step.
4. Introduce 1.5 mL N, N-Dimethylformamide (DMF) into the 3.5 mL PLA solution to give the solution a positive charge. Stir the solution for a further 4 hours at room temperature on a slow spin speed.
5. Load the 3% [w/v] positively charged PLA polymer solution into a glass syringe with an 18- gauge dispenser needle and load into a syringe pump.
6. Attach the positive electrode of the electrospinning rig to the needle.
7. Wrap the electrospinning mandrel with aluminium foil - this allows easy removal of the PLA nanofibers from mandrel once electrospinning is complete. Attach the negative electrode/ground to a mandrel collector.
8. Set the tip of the needle to the mandrel collector to the required distance. In our experiments this distance is set to 21 cm apart.
9. Run the rotating drum at 2000 rpm.
10. Set the potential voltage difference across the electrospinning equipment. Our experiments utilised a 16 kV potential voltage difference by setting the positive electrode to +8 kV at 5mA and the negative electrode to -8 kV at 5 mA.
11. Immediately start the syringe pump and observe the formation of nanofibers on the surface of the mandrel. Run the electrospinning process until desired volume of polymer solution has been successfully spun ( Figure 7A). For this experiment 1 mL of PLA polymer solution was dispensed using a syringe pump flow rate of 80 μL/min.

Production of TEIL frames

1. The PETG circular frames are used to house the nanofibers under tension to allow for culture and handling of the TEIL with minimal disruption to the nanofiber alignment or to the HUVEC cells. Disruption to these components can lead to improper alignment and damage to the HUVEC layer prior to flow experimentation.
2. Download the .STL file from the Keele University data repository. ^36^
3. Upload the file onto the chosen FDM (fused deposition modelling) slicer software such as the open source UltiMaker Cura software ( https://ultimaker.com/software/ultimaker-cura/).
4. Orientate the model so the top surface is touching the build plate and the frame legs are pointing upward (this ensures the flattest possible surface for the interface of the frame and nanofiber mesh)
5. Slice the model using the tuned settings for PETG filament.
6. Save the corresponding.GCODE file to a USB/SD card and transfer to the 3D printer.
7. After printing, allow at least 20 minutes for the build plate and part to cool to room temperature before removing the part from the printer. This prevents the frame from warping, altering the dimensions of the frame.
8. Remove the part, and lightly brush away any stray filament/stringing from the printed part.

Transfer of nanofibers to TEIL frame

Nanofibers are held in an aligned position by transferring them onto the TEIL PETG frames and using biocompatible UV-cured resin as a fixative.

1. Remove the nanofibers deposited on aluminium foil sheet from the mandrel and place onto a flat surface. Use masking tape to secure it to the surface under tension ( Figure 7B). Suspend a 405 nm UV light source above the foil.
2. Apply a very thin layer of biocompatible DSI Tray resin (or other tested biocompatible resin) on the topmost surface of the TEIL PETG frame using a fine paintbrush ( Figure 7C).
3. Place the painted frame face down onto the nanofibers ensuring the larger legs are parallel to the direction of nanofiber alignment ( Figure 7D). This acts as a guide for subsequent positioning of the TEIL in the correct orientation in the microfluidics chip.
4. Apply pressure to the legs of the frame for a minimum of 10-15 seconds under the UV light ( Figure 7D).
5. Repeat steps 4-7 for all TEIL frames. Cut between frames to separate into individual samples.
6. Allow the resin to cure for a further 10 minutes under UV light ( Figure 7D).
7. Use a razor blade gently scrape away excess nanofibers from the outside of the frames ( Figure 7E).
8. Spray all the frames with 70% Industrial Methylated Spirits (IMS; Figure 7E). This is done to reduce nanofiber disturbance or tearing when peeling the TEIL frame from the foil.
9. Use forceps to hold the small legs of the frame.
10. Pull the frame off the foil in the direction of the nanofibers to leave the nanofibers exposed on the surface of the frame ( Figure 7F).
11. Check quality of the nanofibers from the lifted frame to ensure that no holes have appeared in the mesh following processing. Additionally ensure resin fixative has not migrated inward from the edges of the frame.
12. Transfer the Nanofiber-coated frames to 6-well plates with the legs of the frames in contact with the bottom surface of the well plate.
13. Submerge the frames in 70% IMS overnight at room temperature to sterilise them.
14. Remove the IMS and wash the TEIL frames with sterile PBS.
15. Remove the PBS and UV sterilise the frames 3 times before transferring to a new sterile 6-well plate.
16. Place the well plate into a laminar flow hood and keep the lid ajar to allow the nanofibers to dry (they will transition from a transparent appearance when wet to white and semi translucent colour when dry).
17. Coat the fibres with a sterile human fibronectin solution (1 μg/mL dissolved in PBS) for 24 hours at room temperature.
18. Allow the nanofibers to dry in the laminar flow hood before use in the HUVEC seeding process.

Establishment of 2D primary HUVEC culture

Human umbilical vein endothelial cells (HUVECs) were cultured in 2D in cell culture flasks as per the supplier’s recommendations. HCASMCs were cultured in Medium 200 supplemented with low serum growth supplement (LSGS). All cells were grown in a humidified incubator at 37°C and 5% CO _2_. Media was changed every 48-72 hours until the cells were 70% confluent and then changed every 24 hours until confluent. HUVECs did not exceed passage 5.

2.3.3 Construction of TEIL (Video 4)

Prior to trypsinisation and seeding of the HUVECs onto the nanofiber frames, cells are labelled using a cell dye. CellTrace (Fisher Scientific, Loughborough, UK) is a cytosol stain than can retain fluorescence after multiple generations of cells. Fluorescently staining the HUVECs is a key quality assurance step to important to step to ensure correct seeding and growth of the cells during the culture period prior to the onset of experimentation.

**Figure 8.**
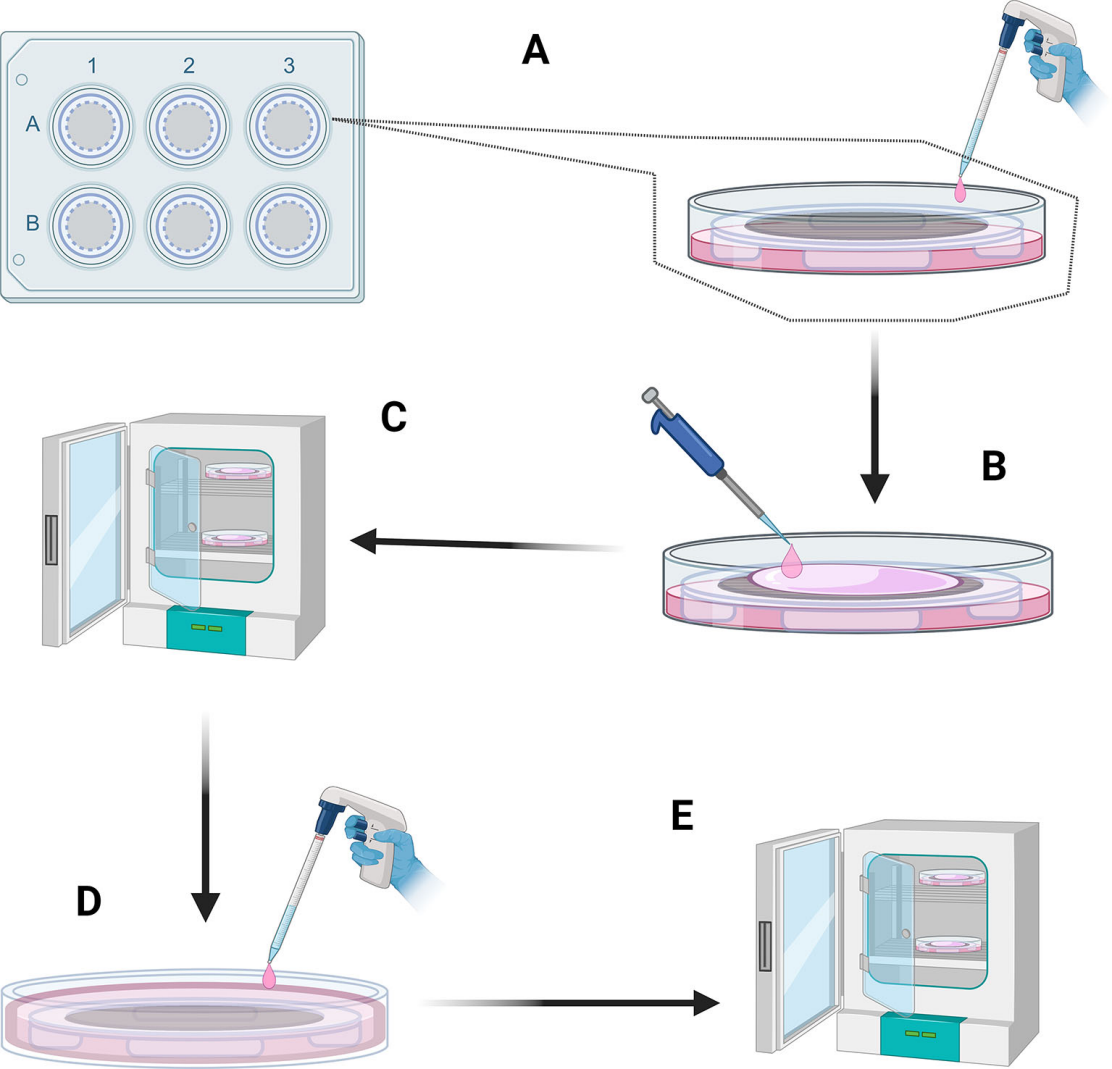
Production of the TEIL. (A) Use supplemented Medium 200 to pre-wet the frames and to provide a reservoir of media in contact with the bottom of the nanofibre frames. (B) Add a HUVEC-containing solution to allow for attachment of these cells to the frames (C) place in incubator and allow HUVECs to attach (D) Add additional supplemented Medium 200 to submerge the frames in this solution (E) Culture the cells in an incubator for 10 days with regular media changes. Figure created with BioRender.com.

1. Prepare a 5 μM CellTrace solution in sterile PBS. This solution is prewarmed to 37°C in a water bath before adding to the washed HUVECs in the cell culture flask.
2. The labelled HUVECs are placed in an incubator at 37°C for 30 minutes CellTrace as per the manufacturer’s recommendations.
3. The CellTrace solution was then aspirated, and fresh Medium 200 supplemented with LSGS was added to the flask and agitated for 5 minutes to quench any remaining extracellular dye. Place pre-coated nanofiber frames in 6-well plates in a sterile hood.
4. Pre wet the surface of the nanofiber frame with 1 mL LSGS-supplemented medium 200.
5. Add an additional 2 mL of supplemented medium 200 to the bottom of the well, ensuring that the media is contact with the bottom of the nanofiber mesh ( Figure 8A).
6. Use the pipette tip to lift the nanofiber frames at the edge to tilt them to remove any air bubbles trapped under the nanofiber mesh. Ensure that the pipette tip does not touch the nanofiber mesh.
7. Prepare HUVECs at a density of 1×10 ^6^ cells/mL in LSGS-supplemented media.
8. Seed 300 μL of this HUVEC solution (2×10 ^5^ cells) onto the surface of the nanofibers using a pipette in a spiral motion. Start in the centre of the nanofiber mesh and working outward to the surrounding frame ( Figure 8B).
9. Place the frames in a cell culture incubator for 30 minutes to allow the HUVECs to settle and lightly adhere to the nanofiber mesh ( Figure 8C).
10. Remove the frames from the incubator and place back in the sterile laminar flow hood.
11. Add 3 mL of LSGS-supplemented media to the well until the level of the media is above the top of the frames ( Figure 8D).
12. Check the TEIL with a fluorescence microscope using an excitation wavelength of 358 nm, and an emission wavelength of 461 nm to visualise the HUVECs. This step ensures endothelial cell adhesion and coverage on the nanofiber mesh.
13. Incubate the TEIL in a cell culture incubator ( Figure 8E). Change media every 48 hours until day 10 of culture. Maintaining sterility of the sample, check the coverage of the TEIL using a fluorescence microscope to ensure correct cell alignment and proliferation on the surface of the TEIL.

Production of the TEML (Video 5)

Establishment of 2D Human Coronary Artery Smooth Muscle Cells (HCASMCs) culture

Human Coronary Artery Smooth Muscle Cells (HCASMCs) culture were initially cultured cell culture flasks per the supplier’s recommendations. HCASMCs were cultured in Medium 231 supplemented with Smooth Muscle Growth Supplement (SMGS). All cells were grown in a humidified incubator at 37°C and 5% CO _2_. Media was changed every 48-72 hours until the cells were 70% confluent and then changed every 24 hours until confluent. HCASMCs did not exceed passage 5.

Sterile filter paper frames for the TEML construct

Filter paper frames are used to allow the TEML hydrogels to take and maintain the correct shape for perfusion. 1×1 cm square frames templates are printed onto filter paper and cut out by hand using a razor blade on a dedicated cutting board, or using a laser engraver/cutter to reduce variation. The template for these frames can be downloaded from the Keele University data repository. ^36^ Once cut the frames should be sterilised by autoclaving using a standard autoclave sterilization program prior to use.

Construction of 3D TEML (Video 5)

The medial layers are constructed by seeding HCASMCs within a type I collagen hydrogel (6 mg/mL) at a final cell seeding density of 5×10 ^5^ cells/mL. After gelation, TEML constructs are compressed to increase substrate stiffness and collagen concentration.

Preparation of biomimetic TEML hydrogels

**Figure 9.**
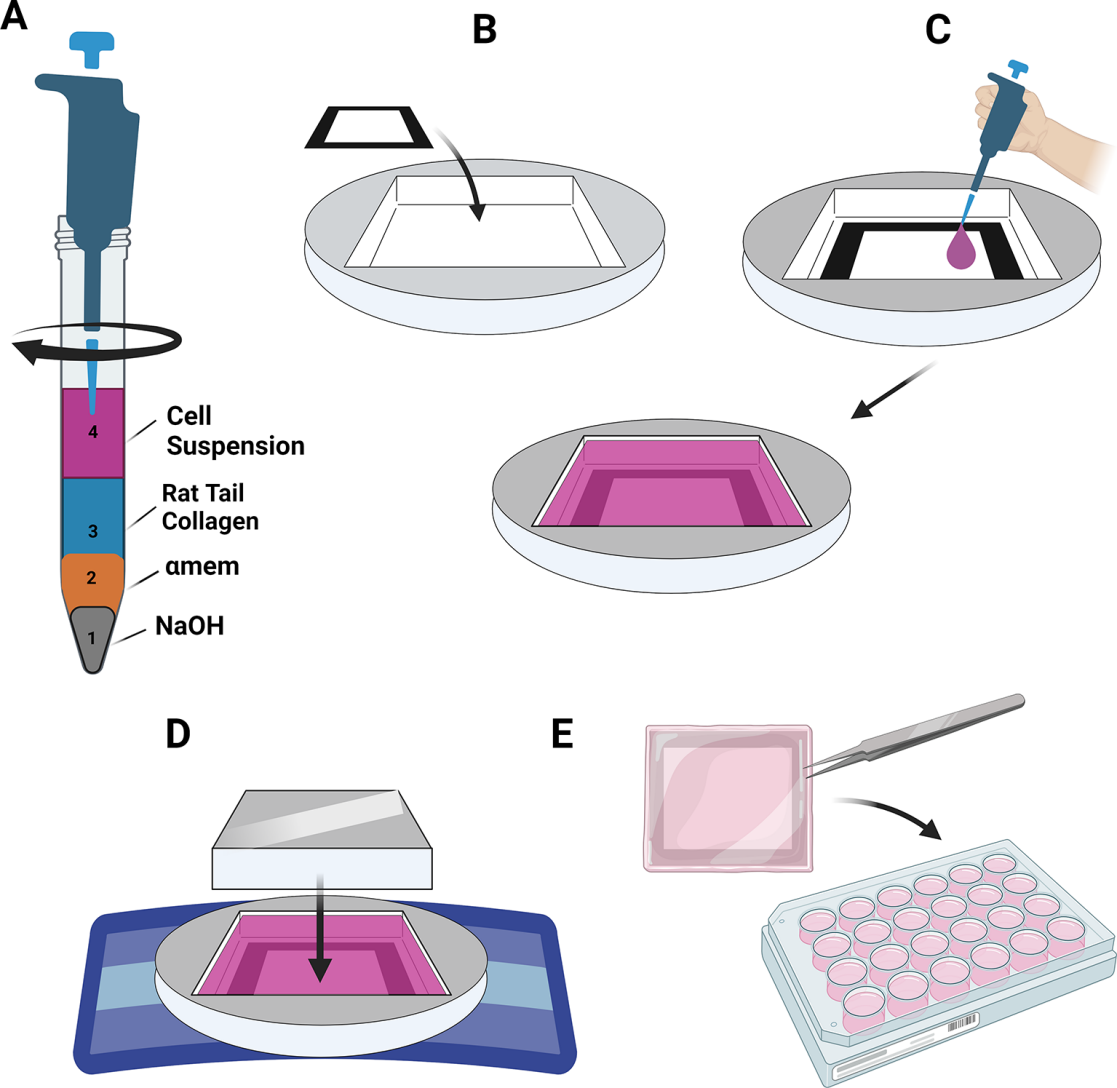
Production of the TEML. Figure created with BioRender.com. (A) Mix the components of the medial layer hydrogel in the order shown (B) Set the medial layer hydrogel into a filter paper frame within stainless steel moulds contained in a sterile petri dish (C) starting around the edges, dispense the hydrogel mixture over the surface of the filter paper frame. Leave to set in an incubator for at least 40 minutes. (D) Use a stainless steel weigh to compress the medial layer hydrogel for 5 minutes when placed atop a nylon mesh on sterile paper towels. (E) Transfer the compressed medial layer hydrogel into 24-well plates containing supplemented HCASMC media for 6 days. Figure created with BioRender.com.

1. Hydrogel reagents are pre-chilled and kept on ice to prevent unwanted gelation during the 3D cell scaffold preparation. Place a sterile rat tail type I collagen, 10 x αMEM (Minimum Essential Medium Alpha), 1N NaOH and a 15 mL falcon tube on ice in a sterile flow hood.
2. Calculate the total hydrogel volume required. Each hydrogel made requires a minimum of 1 mL of final solution. It is advised to make up 1 mL additional hydrogel solution to allow tracking of gelation.
3. Use the following formulas to calculate the required volumes of each of the component solutions:
  • Collagen volume = (Desired concentration (6 mg/mL)/stock concentration) × total volume
  • 10 × αMEM volume = Final volume ÷ 10
  • 1N NaOH solution volume = Collagen volume × 0.023
  • Cell suspension volume = Total volume – (a + b + c)
4. Calculate required HCASMC density for the calculated cell suspension volume to provide a final cell density of 5×10 ^5^ cells/mL
5. Required Cell density (Cells/mL) = (Total hydrogel volume × final cell density (5×10 ^5^ cells/mL))/Cell Suspension Volume (mL)
6. Collect HCASMC cells from cell culture flask by trypsinisation. Neutralise trypsin and collect into a small volume of Medium 231 for cell counting. Resuspend to the requisite HCASMC density calculated in step 4.
7. Add solutions to the falcon tube in order indicated in Figure 9A (smallest to largest volumes). Use slow and steady pipetting motion to limit bubble formation.
8. Using the pipette tip, thoroughly mix the hydrogel solution in a steady and slow manner to reduce bubble formation.

Compression of TEML hydrogels

1. Using forceps, transfer pre-cut 1 × 1 cm square filter paper frames into plastic or stainless- steel moulds ( Figure 9B; frame templates are available in the Keele University data repository. ^36^ Dual moulds have a dimension of 2 x 1 cm and single moulds have a dimension of 1 × 1 cm.
2. Dispense 500 μL of the hydrogel solution into the mould. Dispense solution gradually, starting around the edges to ensure good cohesion between the hydrogel and frame, before working toward the centre of the frame ( Figure 9C).
3. Gently transfer the petri dish into an incubator at 37°C and 5% CO _2_ for a minimum of 40 minutes to allow complete gelation of the hydrogel ( Figure 9D). The leftover hydrogel remaining in the falcon tube can be used to guide if further gelation time is needed.
4. While waiting for gelation, place folded sterile paper towels into the sterile hood and then place the nylon fabric on top of the paper towels ( Figure 9E).
5. Once gel has formed, using forceps carefully remove the TEML and mould from the petri dish and place onto the sterile nylon fabric ( Figure 9E).
6. Place the stainless-steel weight onto the TEML and wait 5 minutes to allow excess water to be absorbed into the paper towels ( Figure 9E).
7. Remove the weight and mould from the compressed TEML. Using fine dissection scissors cut any excess gel between frames away to allow each individual gels to be isolated individually.
8. Transfer each TEML into 24-well plates using sterilised forceps being careful to only grip the filter paper frame ( Figure 9F).
9. Cover the TEML with SMGS-supplemented medium 231 containing 50 μg/mL ascorbic acid (ASC) and 50 μM DETA-NONOate.
10. Culture TEML samples for 6 days before use in flow experiments. Change media every 24 hours with fresh supplemented media.

2.3.4 Production of the mechanical injury press

To induce injury, a method has been developed to ensure reproducible mechanical injury to the human arterial construct. The mechanical injury press (MIP) is a device that introduces needle punctures to the TEAC, positioned in the centre of every channel to produce an *in vitro* thrombosis model. The device is designed to use the minimal number of parts to assemble. The MIP consists of 3, 3D printed parts and 7 bought parts. The MIP can reproducibly create injury of correct size, location, depth and angle thus limiting variation between experiments ( Section 3.2)

3D printed parts

**Figure 10.**
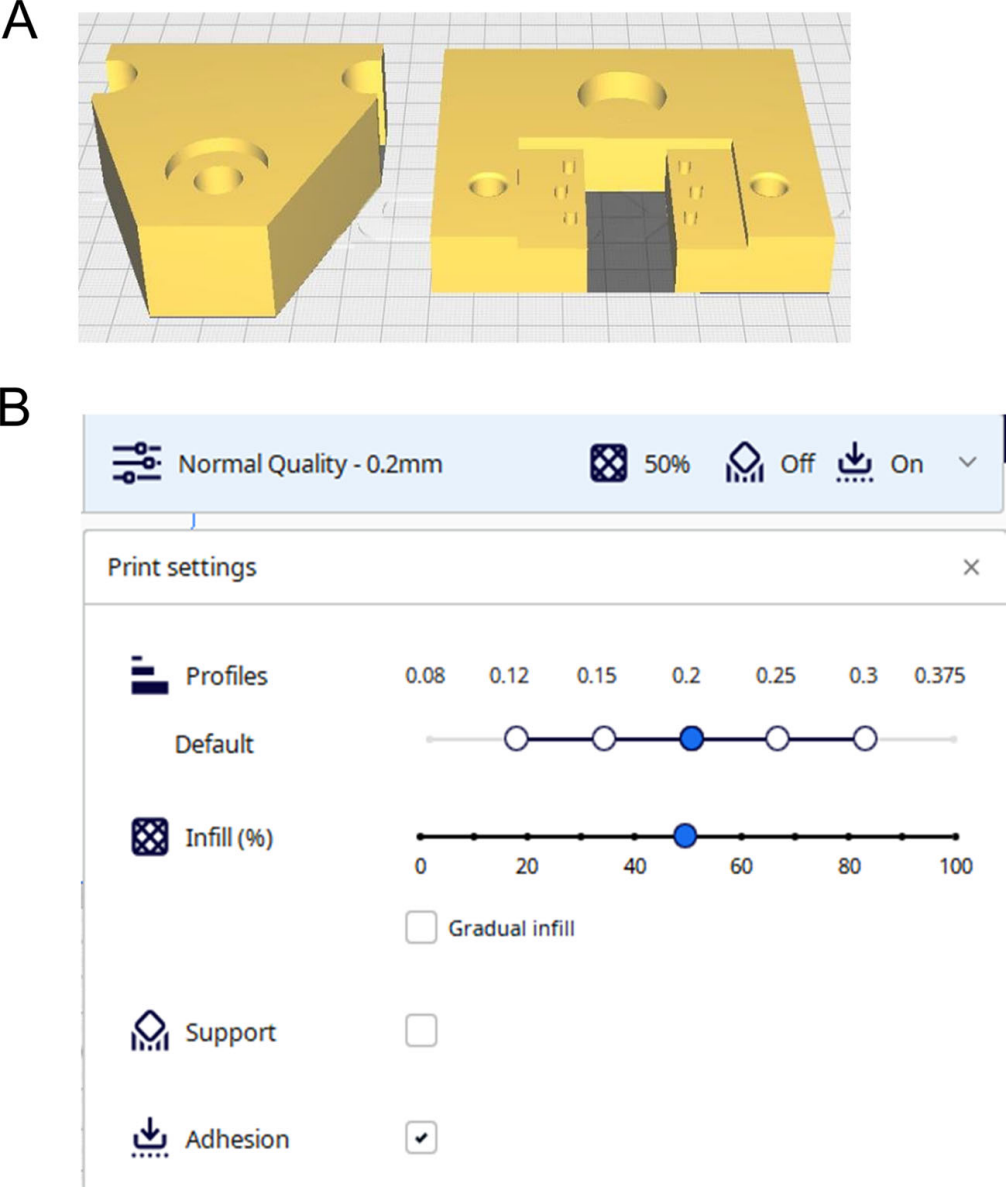
3D Printing set up for components of the mechanical injury press.

1. Download all components.STL files from the Keele University data repository. ^36^
2. Load your FDM 3D printer’s slicer programme.
3. Upload the .STL files to the slicer software and orient the parts correctly on the build surface ( Figure 10A).
4. Set the printing layer height to 0.2 mm and ensure moderate and use slow printing speeds for best results ( Figure 10B). The parts require no supports.
5. Slice the models to a. GCODE file and save to a USB/SD card.
6. Insert drive into the 3D printer and load the. GCODE file.
7. Load PLA filament and ensure build platform is correctly levelled and calibrated (accuracy of printer is paramount to produce a MIP that can accurately injure the TEAC).
8. Start the print.
9. Allow the parts to cool sufficiently before removing from the build plate.

Assembly of the MIP

**Figure 11.**
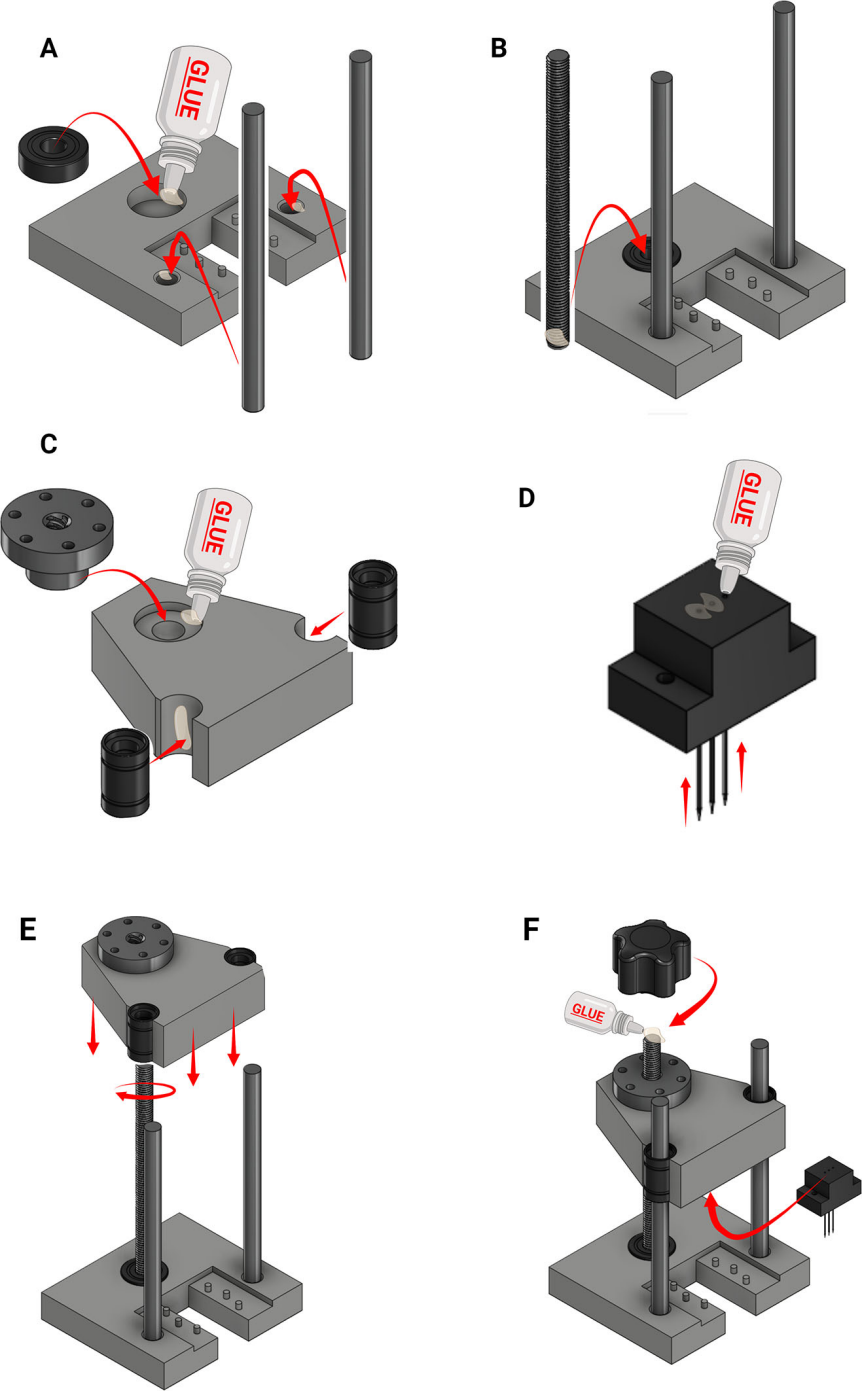
Production of the mechanical injury press. (A) Glue linear rods and bearing into position on the 3D printed base (B) Secure the threaded rod into the bearing. (C) Secure the threaded nut and linear bearings into the actuator part (D) Secure 26-gauge needles into the needle holder with glue. (E) Use the linear rods to position the actuator above the microfluidic chip positioner. (F) Lower the actuator and attach a knob to the top of the threaded role. Screw the needle holder into the actuator. Figure created with BioRender.com.

1. Add super glue to the inner walls of the linear rod holes and bearing hole located on the microfluidic chip positioner ( Figure 11A). Other glues with equivalent bonding properties could also be used.
2. Secure the linear rods and bearing into position ensuring they are accurately positioned and allow to dry before proceeding ( Figure 11A).
3. The M8 threaded rod can be fastened or glued in place depending on the bearing used ( Figure 11B).
  • To glue into place: add a small amount of super glue to the outer diameter of the threaded rod at the bottom end. Insert the rod into the bearing and ensure it is positioned completely perpendicular to the base. DO NOT glue the rod to the 3D printed part.
  • To fasten in place: Loosen the bolt of the bearing. Insert the threaded rod into the bearing whilst ensuring it doesn’t contact the 3D printed part. Tighten the bearing bolt to secure the threaded rods in place.
4. Use super glue to secure the threaded nut to the actuator part ( Figure 11C).
5. Use super glue to secure the linear bearings into actuator part ( Figure 11C).
6. Insert 26-gauge needles to the needle holder. Apply super glue to the holes located at the top of the part to secure the needles in place ( Figure 11D).
7. Position the assembled actuator above the microfluidics chip positioner using the linear rods as a guide ( Figure 11E).
8. Hold the actuator in place and rotate the threaded rod into the actuator nut to slowly lower the actuator in place ( Figure 11E).
9. Once the actuator is lowered sufficiently to expose the top of the threaded rod, use super glue to secure a knob to the top of the threaded rod ( Figure 11F).
10. Use two small screws to secure the needle holder into the actuator ( Figure 11F).
11. Rotate the knob to lower and raise the actuator to its limits, ensuring that the actuator moves in a level manner.

2.3.5 Setting up the humanised in vitro thrombosis model (Video 6)

By incorporating the TEAC into the microfluidics system, we can introduce flow of human blood over the construct to replicate *in vivo* conditions within the vasculature. Flow parameters can be adjusted to achieve pathological or physiological flow conditions seen in the native artery. Furthermore, by collecting blood that assess through the chamber, these post-flow samples can be further assessed to further examine activation of platelets, and the coagulation and fibrinolytic systems.

Citrated human blood samples are collected on the day of the experiment from volunteers who have provided written, informed consent. Platelets collected from human donors are labelled with a FITC-CFSE stain for real time assessment of clot formation under a fluorescent microscope. The TEAC can be used with or without injury depending on the experimental study.

Preparation of human blood

**Figure 12.**
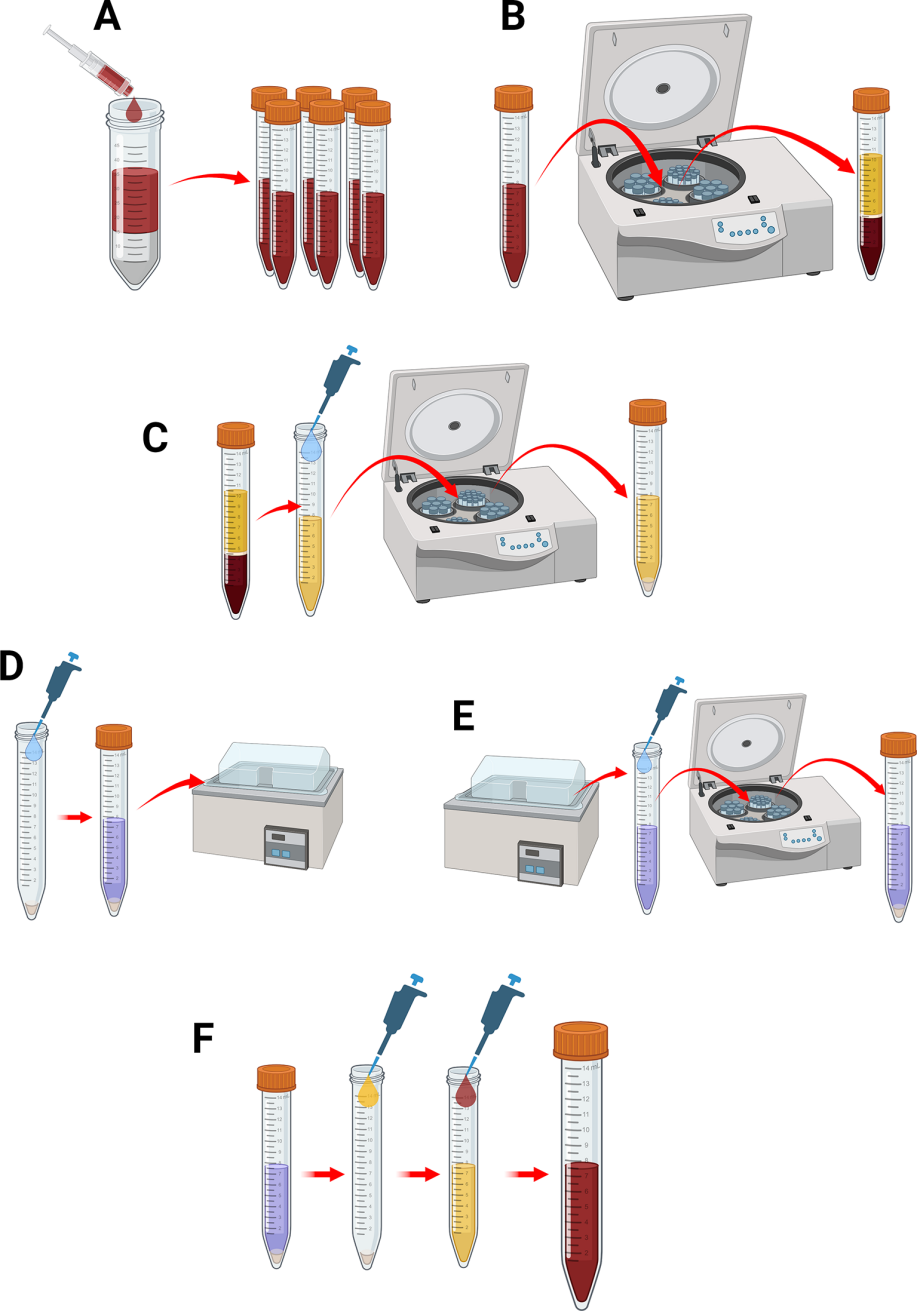
Preparation of whole human blood with CFSE-labelled platelets. (A) Collect whole human blood into sodium citrate (B) Centrifuge to separate blood components (C) Aspirate PRP and treat with apyrase. Centrifuge PRP to produce a platelet pellet. Retain the packed red blood cells and buffy coat for later use. (D) Retain the platelet poor plasma (PPP) and resuspend the platelet pellet into supplemented HBS containing CFSE and incubate (E) Add ACD and prostacyclin and centrifuge sample to recollect the labelled platelet pellet (F) Resuspend the labelled platelet pellet in autologous PPP, and then add back retained buffy coat and packed red blood cells to produce whole human blood with CFSE-labelled platelets. Figure created with BioRender.com.

1. Prior to taking blood on the day of the experiment. Make up a supplemented HEPES-buffered saline (HBS; pH 7.4, 145 mM NaCl, 10 mM HEPES, 10 mM d-glucose, 5 mM KCl, and 1 mM MgSO _4_) by taking the stock solution and adding:
  • 10 mM glucose
  • 0.1% [w/v] bovine serum albumin (BSA)
  • 0.1 U/mL apyrase
  • 200 μM CaCl _2_
2. Blood is collected by venipuncture and mixed with one part 3.8% [w/v] sodium citrate solution to 9 parts whole blood ( Figure 12A).
3. Citrated blood is centrifuged at 700 × g for 8 minutes with soft descent setting enabled to separate the blood into its constituents ( Figure 12B).
4. Aspirate the platelet rich plasma (PRP) into a new 15 mL falcon tube and add 0.1 U/mL apyrase ( Figure 12C). Retain the Buffy Coat and packed red blood cells for later use.
5. Place the PRP into the centrifuge at 350 × g for 20 minutes with soft decent setting enabled. This will yield platelet poor plasma (PPP) and a platelet pellet ( Figure 12C).
6. Aspirate the PPP and retain in a new, sterile 15 mL falcon tube.
7. Resuspend the platelet pellet into 5 mL of supplemented HBS solution and introduce CFSE fluorescent cell stain at a final concentration of 8 μM ( Figure 12D).
8. Place the falcon tube into a water bath at 37 ^o^C for 20 minutes to allow labelling of the platelets ( Figure 12D).
9. Remove the falcon tube from the water bath and add 300 nM prostacyclin and 10% [v/v] ACD to the platelet solution ( Figure 12E).
10. Centrifuge platelet solution at 350 x g for 20 minutes, this will yield a CFSE-stained platelet pellet ( Figure 12E).
11. Remove the HBS solution and resuspend the platelet pellet in its PPP ( Figure 12F).
12. Once thoroughly mixed, reintroduce the blood supernatant into the PRP, and then add this back to the stored buffy coat and packed red blood cells to reconstitute whole blood. Slowly and gently invert sample a minimum of 6 times to allow thorough mixing of the blood ( Figure 12F).

Set up and running of the humanised *in vitro* thrombosis model

**Figure 13.**
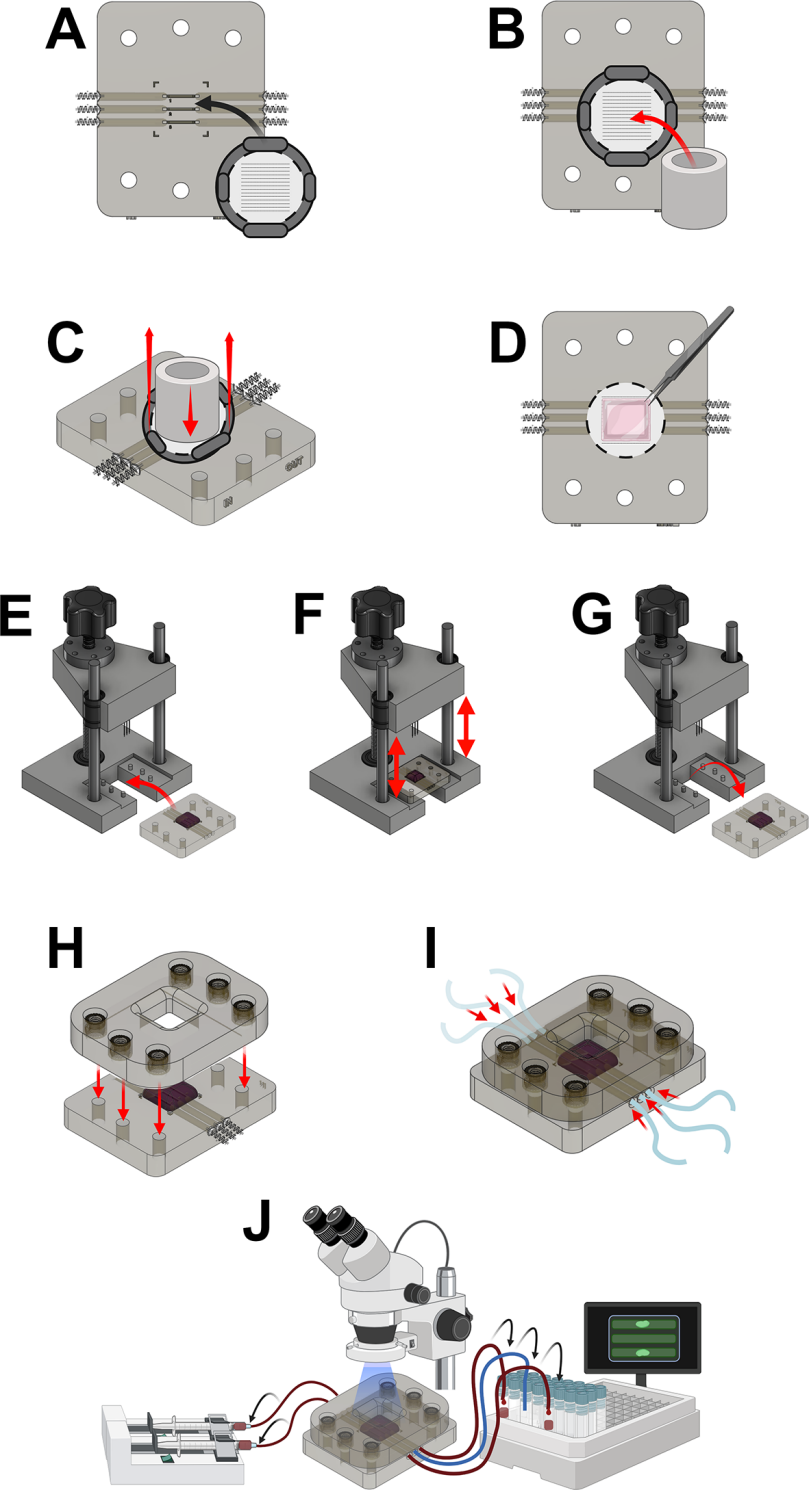
Setting up and running the humanised *in vitro* thrombosis model. (A) Transfer the TEIL onto the surface of the microfluidic chip (B) Place the cutter tool onto the TEIL with the sharp edge pointing downwards (C) Pull the nanofibre frame up whilst producing downward pressure through the cutting tool to sever the nanofibers (D) Place the TEML on top of the TEIL using the guide marks on the microfluidic chip (E) Place the microfluidic chip onto the positioner in the mechanical injury press (F) puncture TEIL by lowering the needle block of the mechanical injury press onto the TEAC (G) remove the injured TEAC from the mechanical injury press (H) Screw the compression bracket onto the microfluidic chip to produce the completed thrombus-on-a-chip model (I) Attaching tubing to the microfluidic chip and connect to a syringe pump containing whole human blood with CFSE-labelled platelets (J) Place under a fluorescent microscopy and initiate perfusion at the desired flow parameters. Monitor thrombus formation in real time using the fluorescence microscope whilst collecting post-flow blood samples into eppendorfs containing ACD. Figure created with BioRender.com.

1. Place the microfluidics chip onto a flat surface.
2. Remove the TEIL from the incubator and place it face down onto the microfluidic channels ( Figure 13A).
3. Place the 3D printer TEIL cutter with the sharp edge down onto the TEIL ( Figure 13B).
4. Using your thumb push down on the cutter while simultaneously pulling the TEIL frame up to remove the TEIL lining from the frame ( Figure 13C).
5. Remove the TEML from the incubator and place the construct down on to the TEIL using sterile forceps. Use the guide markings on the microfluidic chip to ensure correct positioning of the TEML construct ( Figure 13D)
6. Transfer the microfluidics chip into the mechanical injury press, positioning it using the holes on the microfluidics chip and the guideposts on the injury press ( Figure 13E).
7. Lower the needles into the TEAC by rotating the knob at the top of the injury press. Lower the needles until resistance is observed and then raise the needles back to the top of the injury press ( Figure 13F).
8. Remove the microfluidics chip from the mechanical injury press ( Figure 13G).
9. Place bolts from underneath the microfluidics chip into the allocated holes and lower the bracket over the microfluidics chip on to the bolts. Tighten the bolts incrementally, in rotation to ensure the bracket is lowered in a level manner. Tighten all bolts until finger tight ( Figure 13H).
10. Secure Fluid Transfer Tygon tubing to all inlet and outlet barbs using a gentle rotating motion ( Figure 13I)
11. Place the thrombosis model under a fluorescence microscope and position the channels using brightfield imaging ( Figure 13J)
12. Re-calcify the required volume of CFSE-labelled human blood and load into the syringe pumps.
13. Attach the top and bottom channel tubing to the syringe pumps ( Figure 13J). Upon occlusion the heightened hydrostatic pressure in the occluded channel forces blood to leach out of the points of damage and through the hydrogel. The middle channel is used as a ‘blow out’ channel to ensure that if thrombotic occlusion occurs in any channel, blood leaking out of the top or bottom channel cannot impact upon the activity in the other channels.
14. [Optional] To collect post-flow blood samples leaving the microfluidics chip, place the outlet tubing of the microfluidics into collection tubes containing ACD ( Figure 13J). These post-flow blood samples leaving the chamber can be analysed directly after the experiment or fixed and platelets assayed later.
15. Switch to the fluorescence setting on the microscope with excitation and emissions of 485 nm and 501 nm, respectively.
16. Start the syringe pumps at the blood flow rates. The flow rates depend on the specific human disease or condition the group is trying to replicate in their experiment. Different conditions may require varying flow rates to accurately model the physiological environment
17. Observe clot formation in real-time using fluorescence microscopy

## 3. Results

### 3.1 Injury model assessment

**Figure 14.**
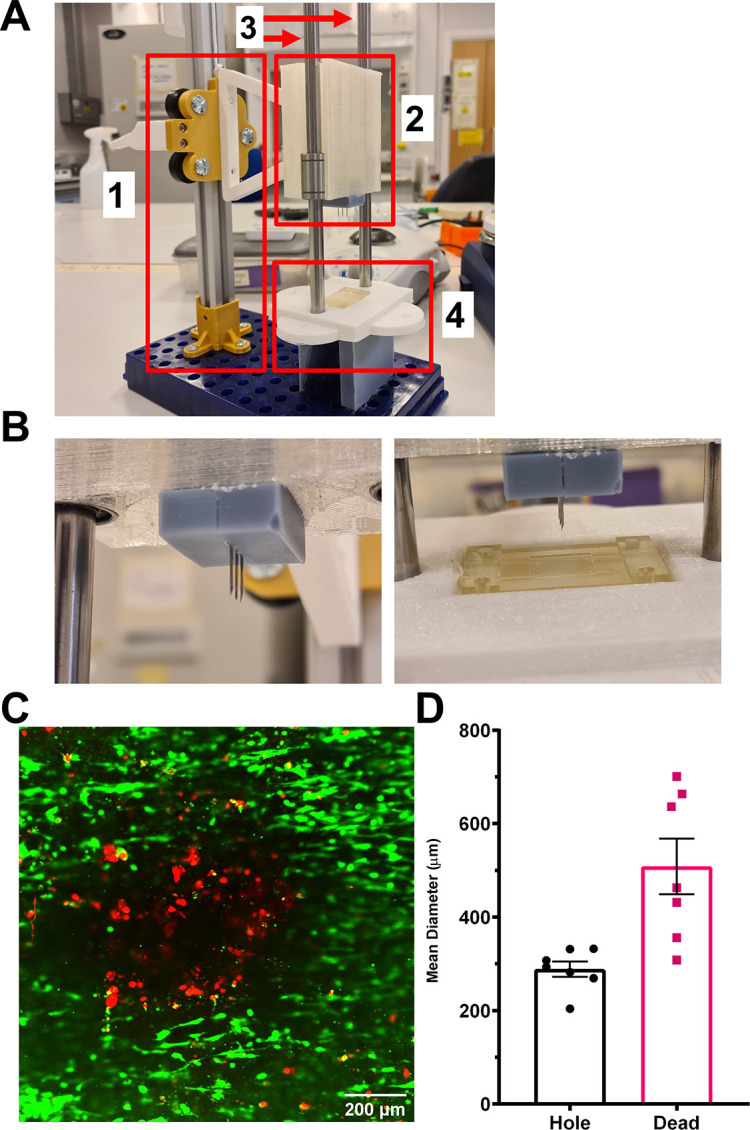
Development and use of a mechanical injury press that can elicit reproducible injuries in the TEAC. (A,B) Design of the mechanical injury press (C,D) Assessment of the reproducibility of arterial injury caused by mechanical puncture. Holes were produced in the top and bottom channel were assessed from 3 independent MI TEACs.

To utilise the TEAC as part of a humanised *in vitro* thrombosis model, a method is required to injure the endothelial lining to reproducibly initiate thrombus formation. Mechanical injury models utilise puncture of the blood vessel wall to initiate endothelial damage and have been used commonly in arterial thrombosis models used to assess *in vivo* thrombus formation. Whilst these provide a more physiologically-relevant mode of activation than FeCl _3_- or laser-induced injuries, the reproducibility of this model is subject to significant variability in the location, depth, and angle of insertion of the needle due to differences in arterial accessibility and inter-operator variance. To assist us in utilising a reproducible mechanical injury model, we have developed a mechanical injury press that can deliver needle punctures to the TEAC in a reproducible manner. The design was initially based on the schematics of a soldering iron press found on Myminifactory.com. The soldering iron holder of the design was replaced with a puncturing attachment and a microfluidic chip holder. The mechanical injury press can be presented in 4 parts; the original press and arm (1 on Figure 14A), the guide head with the needle attachment (2 on Figure 14A) guide rails (3 on Figure 14A) and the microfluidics positioner (4 on Figure 14A; magnified in Figure 14B). The mechanical injury press was assessed for variability of the hole size and size of cell damage it produced in the TEAC construct. This was assessed using live/dead cell staining of the puncture site on the TEAC after use of the mechanical injury press ( Figure 14C). Results showed that the holes made by the mechanical injury press were highly-reproducible with no significant differences between sets (288.3 ± 16.5 μm; n = 7; Figure 14D; as assessed by the largest diameter across the hole in which no stained cells could be observed). Although puncture size was consistent, the zone of endothelial cell death surrounding the puncture site was larger and a little more variable (508.3 ± 55.0 μm). Further experiments will be performed to understand whether the variability in the damage created by the needles is due to localised disruption of nanofibers at the puncture site or due to intercellular signalling causing localised induction of apoptosis. Understanding the mechanisms eliciting this minor variation could allow us to create a less variable injury response that could improve the reproducibility of the elicited thrombotic response. The overall effect is still reliable and can be used to elicit a consistent mechanical injury in the TEAC.

### 3.2 Reproducibility and variability

**Figure 15.**
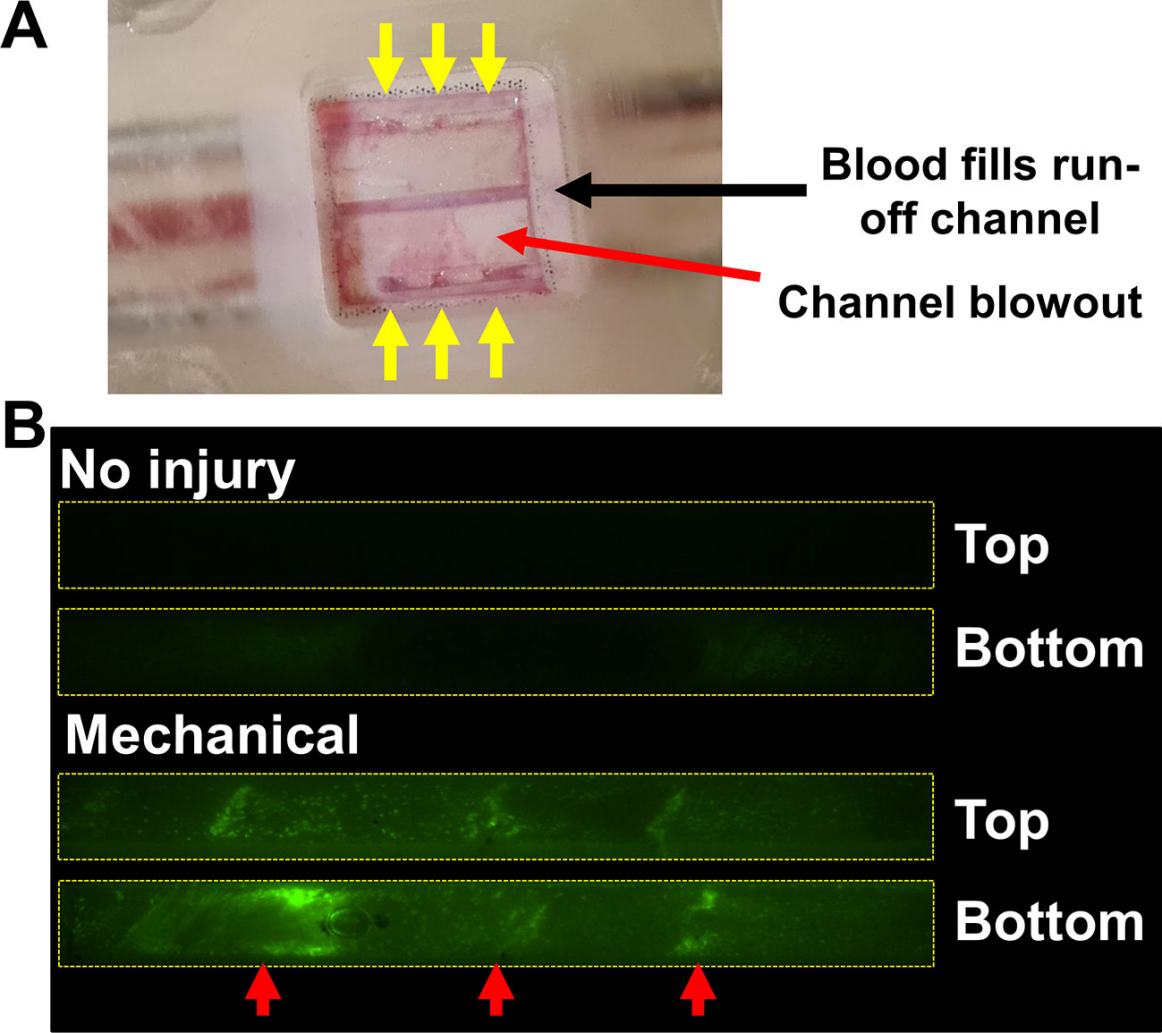
Thrombus formation only occurs in mechanically injured TEAC. (A) Post-perfusion image of TEAC following blowout. Thrombi (identified by accumulation of red blood cells; red arrows) can be observed in the top and bottom channels. Thrombi restrict flow and trigger increased blood pressure that causes leak of blood from the channels through the TEML to the middle channel (B) Fluorescent imaging of uninjured and mechanically-injured TEACs during perfusion with whole human blood with CFSE-labelled platelets. No accumulation of CFSE- located platelets observed after 10 minutes of perfusion in uninjured TEAC. In contrast, mechanical injury invokes thrombotic responses at sites of injury (red arrows). The positions of the top and bottom channels on the microfluidic chip are indicated.

Having developed the microfluidic chamber, the TEAC and a reliable injury model, experiments were performed to create a complete humanised *in vitro* thrombosis model to assess the effect of mechanical on the thrombotic response observed under physiological flow conditions. The microfluidics chip was assembled with the TEAC and connected to a syringe pump as described above and as shown in video 6. Re-calcified whole human blood with CFSE-labelled platelets was perfused through the microfluidics at 160 μL/min for 10 minutes or until channel blowout was visually detected. This blood flow rate was calculated to provide shear stress of approximately 24 dynes/cm ^2^, which is in the physiological range for arteries. The observation.of blowout in some channels appeared to be caused by channel occlusion ( Figure 15A). Blowout is defined as human blood escaping the intended top or bottom channels due to the increased blood pressure after channel occlusion. Similar phenomena have been previously reported in other 2D *in vitro* thrombosis models. ^26^ Our initial trials identified that blowout could be isolated to the top or bottom channel by leaving the central channel free to capture blood leaving the affected channel and funnelling it to the outlet. Therefore, the central channel was utilised as a pressure-relief channel, which helped us perfuse the other two channels independently by preventing blood from reaching the other. Perfusion was halted as soon as blowout was identified to further protect against bleed-through between channels.

Reproducibility of the thrombotic response was performed using an analysis of previously performed assessment of the performance of the uninjured and mechanically injured construct. ^37^ (Ranjbar *et al*., Manuscript in preparation). Thrombus formation is not observed in control in which no injury was induced in the flow channels prior to the onset of blood flow found that there was no significant accumulation of platelets in any region of these channels over the 10 minutes of flow demonstrating that the intimal lining was effective at preventing thrombus formation in uninjured samples ( Figure 15B). This is consistent with our previous findings that showed that the uninjured TEAC protects against thrombus formation. ^33, 34^ There was no significant difference in the mean fluorescence found in both the top (4.6 ± 0.9 arbitrary units) and bottom channel (4.9 ± 1.1 arbitrary units) of the uninjured channels (n = 5 chips; P = 0.59; Figure 15B). In contrast, in TEACs subjected to mechanical injury 3 platelet-rich thrombi can be observe to form at the site of each of the 3 puncture injuries in the top and bottom follow channel ( Figure 15B; red arrows). This pattern was seen in all experiments and demonstrated that thrombi reliably form at sites of mechanical injury. To assess inter-channel reproducibility of the thrombotic response observed in our mechanical injury model, we compared the mean fluorescence observed after 5 mins of perfusion of the top and bottom channels across different TEACs. Results showed no significant difference in the mean pixel fluorescence seen in the top channel (10.5 ± 2.9 arbitrary units) compared to that of the bottom channel (12.8 ± 4.1 arbitrary unit, p = 0.23 n = 5 chips) at the end of the perfusion period. These results indicate that although there is some variation in fluorescence observed between DiOC6-labelled blood samples prepared from different donors, there is satisfactory inter-channel reproducibility of the thrombotic response on the same TEAC, such that they can be used to make a valid assessment of the effect of anti-thrombotic therapies.

Post-perfusion assessment of the flow channel using optical coherence tomography

**Figure 16.**
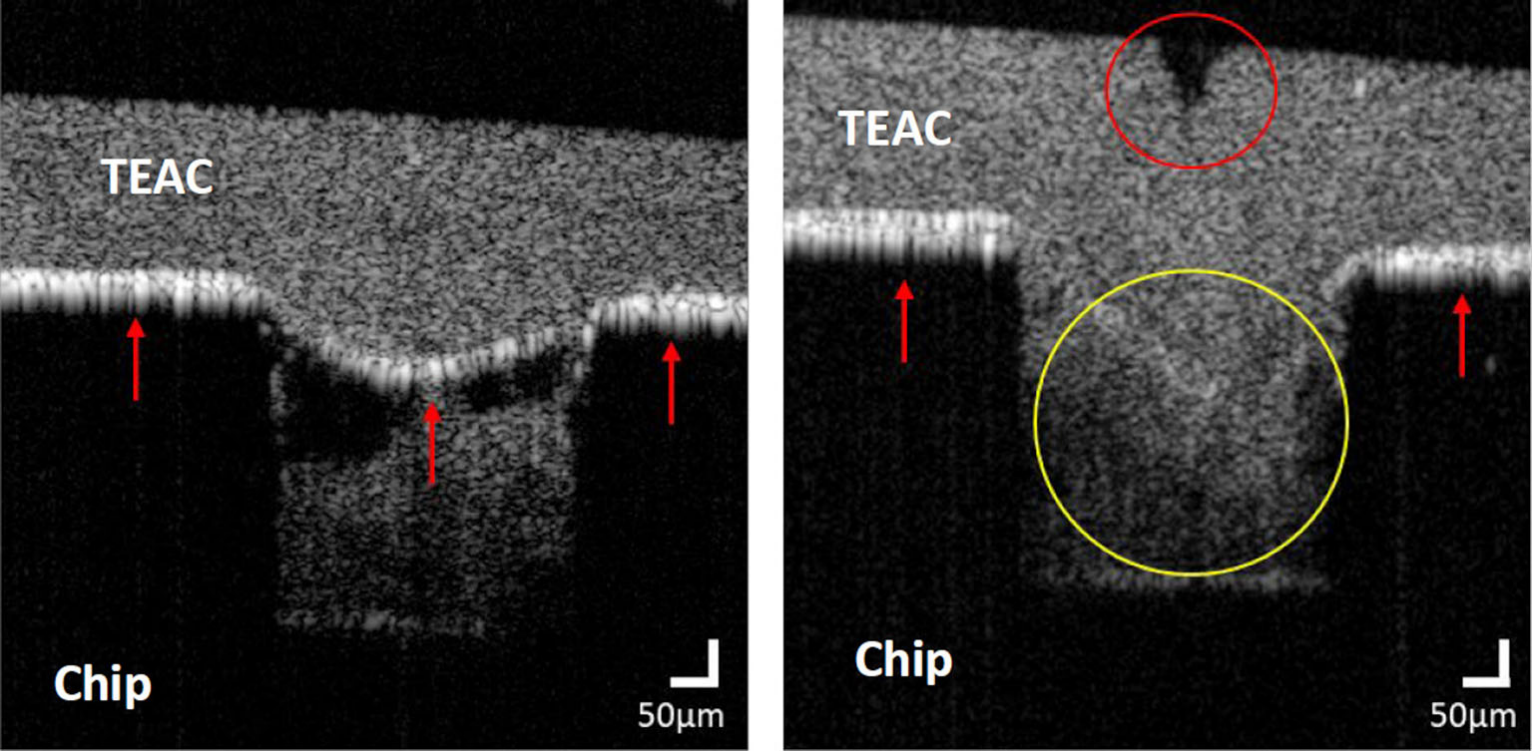
OCT imaging of an uninjured (Left panel) or punctured (Right panel) segment of the TEAC in a perfused thrombus-on-a-chip model. Red arrows highlight the position of the intimal lining as identified by the higher back reflectance of this layer compared to the overlying medial layer. The red circle highlights the indentation of the intimal surface that shows the position of the puncture site and the small bowing of the gel into the flow channel. The yellow circle highlights the disruption of the intimal surface seen at the puncture site.

Following injury of the TEAC and its perfusion with DiOC6-labelled human blood, we performed pilot studies to assess the morphology of the flow channel and TEAC using OCT imaging. Images showed the ability of the OCT to capture the different components of the model, including the microfluidic channels, TEIL, TEML, coverslip and, more importantly, the area of mechanical injury ( Figure 16). Images in uninjured sections observed a small bowing of the TEAC into the channel when compressed using the bracket (Left panel, Figure 16); this may be due to the gel’s stiffness not fully supporting the weight of the unsupported gel. Future enhancements in the mechanical properties of the gel would be expected to further reduce the impact of this bowing on laminar flow across the construct. As this reduces the depth of the channel we assessed the impact on the elicited shear rates in the centre of the channel where thrombi are generated, the depth of the channel was measured from the base of the channel to the peak of the bowed hydrogel (252 μm ± 4 μm, n = 5). The channel width of the flow channel was also measured along the bottom of the channel (360 μm ± 8 μm, n = 5 puncture sites). Using these data, blood shear stress was calculated as 24.5 dynes/cm ^2^ using the Darwin Microfluidics calculator, using the set blood flow of 160 μL/min produced by the syringe pumps. This is within the normal range of arterial shear stress and demonstrates that this model system can produce physiological blood flow conditions.

At the point of needle injury, the puncture site of the gel can be generally observed to induce an indentation in the upper surface, alongside a loss of the areas of high back reflectance emitting from the TEIL demonstrating that the puncture has successfully disrupted the endothelial layer (Right panel, Figure 16). Interestingly a build-up of denser (white) areas of a similar intensity below the endothelial layer can be observed in the flow channel at these sites of mechanical puncture. From the confocal imaging data, we would expect this to be due to the accumulation of cells within the thrombus. Further studies will help us to assess whether OCT imaging could provide another method for assessing clot size and structure following mechanical injury.

## 3. Discussion

In this paper we have detailed the successful production of a human thrombus-on-a-chip model, produced through successfully incorporating a human TEAC with functional haemostatic properties within a bespoke 3D printed microfluidic chip to allow perfusion with whole human blood samples under arterial flow conditions. This method provides a humanised drug testing platform for pre-clinical anti-thrombotic therapies. Through selective disruption of the anti-thrombotic properties of the intimal layer and exposure of native human fibrillar collagen and tissue factor in the medial layer. ^7, 33, 34^ This contrasts with many current *in vitro* thrombosis models that use exogenous, non-human sources of collagen and tissue factor, and may lack the presence of an endothelial lining. ^37^ The TEAC demonstrated here provides an effective thrombotic substrate as it can support the activation of haemostatic reactions under physical flow conditions. Further experiments have demonstrated that the thrombotic response evoked is sensitive to prior treatment of the blood samples with clinically-used anti-platelet and anti-coagulant drugs. ^38^ These data demonstrate that the thrombus-on-a-chip model can provide a viable replica of blood clotting in the human body, and as such provides a humanised testing platform for the pre- clinical testing and optimisation of novel anti-thrombotic therapies – which could replace our estimated use of over 5000 animals in this type of research.

This method can also replace the use of arterial thrombosis models to study the process of *in vivo* thrombus formation in mice and other model species. This is commonly performed using a tail bleeding assay in mice in which the tip of the tail is cut and the time taken for bleeding to cease is measured. However this technique is known to give highly variable data and its performance has been difficult to standardize between labs – which significantly increase the number of a animals used in these studies. ^39, 40^ Here, we demonstrate that our mechanical injury press can elicit reliable thrombus formation under physiological flow conditions. The reproducible nature of the puncture wound generated by the mechanical injury press is consistent between samples, thus offering a standardised stimuli for triggering physiological thrombus formation in samples. Additionally, this injury model better replicates the process of blood vessel disruption in the body and therefore removes the problems associated with commonly-used artificial injury modalities used in *in vivo* studies; such as the artefactual, physicochemical activation of thrombus formation seen in FeCl _3_ injury models and the variability in lesion size seen in laser-induced injury models. ^41, 42^ Therefore, this thrombus-on-chip model offers the potential to provide a realistic and reproducible model of human haemostatic function and thus replace the widely-used tail bleeding assay. It can also be utilised as an in vitro assay for fundamental scientific studies assessing thrombus formation and vascular damage. For instance, we have previously used this to assess the tissue engineered arterial construct to assess endothelial progenitor cell homing to sites of vascular damage. ^34^

The use of human thrombus-on-a-chip model could improve the translational success of preclinical trials of new anti-thrombotic therapies in subsequent clinical trials. Firstly, by performing the study with human blood and arterial cells in near-physiological environment, we eliminate the confounding effect of species differences at the outset of the bench-to- bedside process. ^21^ Secondly, we can design studies that assess the impact of drugs across more genetically-diverse populations than most *in vivo* studies, which rely upon the use of inbred strains of mice and other mammalian species. This lack of diversity is sometimes further exacerbated in *in vivo* studies through exclusion of female animals through the choice of the cremaster muscle arteriole as the targeted vessel bed. ^6^ Inequalities in female participation in anti-thrombotic drug testing can lead to harm in clinical practice. ^43^ Through recruiting blood donors we can assess potential differences in drug efficacy and dosages in donors of different ages, sexes and ethnicities (for example). Subsequent subgroup analyses of such data could therefore inform clinical trials to ensure that we ensure clinical application of these drugs are equitably optimised for the widest possible population to ensure the correct balance between effectiveness and adverse bleeding events. Additionally, many anaesthetics used in the study can inhibit platelet function, which through providing a baseline inhibition could overemphasise the degree of inhibition being generated by the trialled treatment. ^44– 46^ Lastly, arterial thrombosis models use the exposure of the subendothelial matrix to elicit thrombus formation, rather than the distinct lipid-rich, calcified, necrotic core of the ruptured atherosclerotic plaque that triggers clotting in heart attacks and strokes. Previous studies have demonstrated that the thrombotic response elicited by human atherosclerotic plaque material differs to that produced by the healthy subendothelial matrix due to differences in their collagen structures. ^47, 48^ A mismatch between the surfaces triggering the thrombotic responses in heart attack and stroke is likely to underlie the failure of many anti-thrombotic agents to translate into effective prevention of atherothrombosis in patients. Recently we have demonstrated that the tissue engineered arterial construct can be modified to incorporate an early-stage atherosclerotic plaque that should help improve the translational capacity of these in vitro models. ^49^ Through providing a more realistic environment in which to study the effect of drug treatments on thrombus formation, we provide a basis to improve the predictive capability of preclinical tests that help to reduce the resources wasted on failed clinical trials.

A key consideration when developing this methodology has been in ensuring that the methodology is as accessible to other groups as possible to ensure that the model is able to be widely adopted across our research community and avoids the 3Rs ‘valley of death’. ^50^ This has included minimising the cost of production of the TEAC and microfluidic chip, ensuring that production tools and materials are widely accessible to other labs, and that that techniques can be easily reproduced in other labs. This has motivated the use of hobbyist 3D printers to produce many components of the system to ensure that the start-up equipment and infrastructure costs for these experiments are minimised and ensure that it can be widely adopted by groups around the world. This offers a significant advantage over current *in vivo* studies that require both the infrastructure to support animal husbandry but also often require expensive and specialist intravital imaging equipment to allow experiments to be performed. Although we have demonstrated the use of fluorescence microscopy to monitor the thrombotic responses, the thrombus-on-a-chip model can also be used to monitor functional readouts that do not require expensive imaging equipment. This includes simply monitoring the rate of flow of blood emerging from the chamber and measuring the latency from onset of perfusion to cessation of flow due to thrombus within the chamber. Alternatively, the blood leaving the chamber can be fixed and assessed for markers of platelet and coagulation cascade activation (Ranjbar *et al*., manuscript in preparation). Additionally, an analysis of the cost of consumable to provide each chip (TEAC and microfluidic chamber) found that they cost less than £25 to produce ( Table 1), which is significantly cheaper than the cost of maintaining breeding colonies of mice and other mammalian species. These properties of the thrombus-on-a-chip model will allow for more equitable access to researchers to the technique demonstrated here.

**Table 1.** The cost of production of each humanised *in vitro* thrombosis chip.

Consumable	Cost per experiment
HUVEC culture media	£6.85
Type I Rat Tail Collagen	£6.00
HCASMC culture media	£3.04
Flow peripherals	£2.31
HCASMCs	£1.50
HUVECs	£1.00
Nanofiber production	£0.50
Microfluidic chip	£0.15
Total Cost	**£21.35**

Whilst the various techniques and tools used have been designed to be accessible as possible, the process of producing and setting up the thrombus-on-a-chip model is non-trivial. To overcome this barrier, we have produced a step-by-step video guide to the production and use of the thrombus-on-a-chip model to aid understanding of the written instruction and ensure that this technique can be replicated effectively in other labs. This is further supported by the distribution of open-source files for the microfluidic chamber and mechanical injury press to ensure that groups have easy access to the components without needing to develop significant expertise in 3D modelling or 3D printing methods. These can be found in the Keele University data repository. ^36^ Our lab is also available to offer advice and support anybody looking to adopt this technique. We will continue to work to further increase the accessibility of the model. This includes aiming to produce constructs either with commercially available nanofibers or without this layer to reduce the need to access this specialized material and to work towards a start-up kit to minimize the production of components to further improve the accessibility of the thrombus-on-a-chip model to labs in universities that lack the facilities and expertise to support uptake of this model. However, these barriers are reduced compared to those faced by groups looking to adopt *in vivo* studies. These include the need to undertake training and supervision required to legally and ethically undertake *in vivo* studies, specialist, expensive animal husbandry centres and intravital microscopy facilities to undertake the experiments, and the time and finances needed to support the maintenance of mouse colonies. When coupled with the poor translational potential of the results obtained from animal studies, there is a compelling case building for labs to adopt 3Rs approaches to thrombosis and haemostasis studies.

## 4. Conclusion

The use of this human thrombus-on-a-chip model incorporating TEACs offers a new methodology that can reduce the use of animals in haemostasis research whilst improving the translational capabilities of preclinical trials of new anti-thrombotic therapies. The methodology provided here provides a viable alternative to both current *in vitro* and *in vivo* thrombosis models that provides an emphasis on replicating the native human environment by utilising human cells throughout to produce the pro- and anti-thrombotic compounds that regulate *in vivo* human thrombus formation. By providing open access to this initial methodology and the developed materials we have worked to remove many of the barriers to uptake of this technique. We hope that the thrombosis and haemostasis community will work with us to further develop these methods to produce an accessible, transferable and widely-adopted technique that will render the use on *in vivo* models obsolete.

## Ethics and consent

This study received approval from the Keele University Research Ethics Committee (Study reference: MH-200152; Approval date: 1 ^st^ December 2020) and was conducted in accordance with the Declaration of Helsinki. Blood was donated by healthy volunteers under written informed consent.

## Data Availability

Data is available from the Keele University Data Repository at https://doi.org/10.21252/ac05-bg65. ^36^ This project contains the following underlying data:
• Template for Filter Paper Frame for TEML hydrogel. A publisher file which can be used as a template to print the filter paper frames used to scaffold the TEML hydrogel. • Microfluidic Chip.zip. This zip file contains.stl files that are used to 3D print the parts for the microfluidic flow chamber. • Mechanical injury press.zip. This zip file contains.stl files that are used to print the original components for the mechanical injury press. The construction of these parts into the mechanical injury press is detailed in this paper. • Nanofibre frame for TEIL culture.zip. This zip file contains.stl files that are used to print the original components for the mechanical injury press • Underlying data.zip contains the original.xlsx, .oib and.jpg data files containing data and analyses for Figures 1, 14, 15 and 16. This project contains the following extended data:
• Extended data – word.zip contains example donor consent forms and volunteer information sheets used in this study. • Extended data – video files 1.zip contains the mp4 files of instructional videos 1,2 and 3 demonstrating Microfluidics printing and post processing, microfluidic peripheral production and TEIL frame production. • Extended data – video files 2.zip contains the mp4 files of instructional videos 4,5 and 6 demonstrating TEIL cell culture, TEML culture and the running of the in vitro thrombosis model. **Playlist for instructional videos (Extended Data)** The instructional videos can be downloaded from the Keele University Data Repository. ^36^ The playlists provide information on where each step can be found in these videos. **Video 1: 3D Printing the Microfluidic flow chip** This video demonstrates how to make the microfluidic flow chamber using a 3D printer using the provided chip model
1. Set up and calibrate printer (0:00) 2. Printing microfluidics chamber (3:00) 3. Cleaning microfluidics chamber (5:20) 4. Post-print curing of microfluidics (10:30) **Video 2: Completing the production of the 3D printed microfluidic chip with peripheral components** This video describes the process of:
1. Microfluidics Inlet/Outlet creation (0:00) 2. Insertion of the microfluidics bracket to produce the viewing window (8:00) **Video 3: Producing aligned nanofiber scaffold for the TEIL** This video will demonstrate how to use an electrospinning rig to produce the aligned PLA nanofibers used as a scaffold for the TEIL.
1. PLA nanofiber solution preparation (0:00) 2. Printing TEIL frames (5:00) 3. Electrospinning PLA nanofibers (5:30) 4. Combining frames and nanofibers (10:40) 5. Fibronectin coating of nanofibers (16:50) **Video 4: Establishing the TEIL Cell culture** This video demonstrates how to coat the aligned PLA nanofibers with fluorescently-labelled HUVEC cells and how to monitor these for confluency
1. Labelling HUVECs (0:00) 2. Making HUVECs seeding solution (6:25) 3. Seeding TEIL frames with labelled HUVECS (10:30) 4. Quality control and feeding (14:45) **Video 5: Producing and culturing the TEML hydrogels** This video will show how to produce compressed 3D collagen hydrogels containing human coronary artery smooth muscle cells
1. Mould preparation (0:00) 2. TEML solution prep (1:40) 3. Seeding moulds (6:50) 4. Compressing TEML (8:30) 5. Cutting and transfer of TEML (10:20) **Video 6: Setting up and running the thrombus-on-a-chip experiments** This video will show you how to use the produced TEIL and TEML layers with the microfluidic chip to produce the complete thrombus-on-a-chip model, and how to utilise this as a humanised in vitro thrombosis model.
1. Prepping work area and equipment (0:00) 2. Transferring TEIL and TEML to microfluidics chip (2:45) 3. Injuring TEAC and preparing microfluidics chamber (5:16) 4. Transferring blood sample to syringe pump (7:50) 5. Connecting inlet to syringe pump and running the experiment under physiological flow conditions (9:15) All data are available under the terms of the Creative Commons Attribution 4.0 International license (CC-BY 4.0)
