# Supplementary material for: A humanised thrombus-on-a-chip model utilising tissue-engineered arterial constructs: A method to reduce and replace mice used in thrombosis and haemostasis research
Source: F1000Res. 2025 Jan 20;14:110. [Version 1] doi: 10.12688/f1000research.158910.1 (PMC11971621; doi:10.12688/f1000research.158910.1)
Supplement: Peer review history

# Reviewer report: Reviewer response for version 1

**DOI:** 10.5256/f1000research.174562.r369850

**Published:** 2025-04-18

**Authors:** Fahima Akther

**Affiliations:** 1 Yale School of Medicine, New Haven, Connecticut, USA

The authors have submitted a paper describing the methodology used to develop a new 3D model for thrombosis-on-chip, which holds promise for reducing reliance on animal models. The concept is compelling, and the methodology is clearly explained in the accompanying video tutorial. However, I found the paper technically challenging and highly ambitious regarding the proposed applications of the model. While the device shows potential to mimic thrombosis in a microfluidic platform, several aspects require clarification and further validation before the manuscript can be considered for indexing.

  

1. Although the paper provides detailed video tutorials outlining the model development and experimental procedures, the bench-to-bedside translation appears challenging. Establishing such a system requires a fully equipped laboratory and personnel with high technical expertise. Moreover, the time required for device fabrication and experimental setup, along with the success rate of the procedure, are critical factors that should be discussed to assess the model’s practical viability as an alternative to animal testing.
2. The authors propose this model as an alternative to both animal models and conventional PDMS-based devices. However, several studies have introduced advanced methodologies with enhanced physiological relevance, such as stretchable microfluidic devices that replicate mechanical strain. It is important to address how this model compares in terms of robustness and physiological mimicry under such conditions.

For example: Gu X et al [2019 9Ref-1)] https://www.nature.com/articles/s41598-019-43902-3

 3. The manuscript mentions the formation of a 3D intimal-medial layer, but evidence confirming the three-dimensional architecture is lacking. Confocal microscopy images showing distinct layers are recommended to substantiate the formation of the 3D structure. Relevant references include: Akther F et al [2025 (Ref-2)] https://pubs.rsc.org/en/content/articlehtml/2025/lc/d4lc00868e

 Su C et al [2021(Ref-3)] https://pubs.rsc.org/en/content/articlelanding/2021/lc/d1lc00131k

 Zhang X et al [2020(Ref-4)] https://www.nature.com/articles/s41467-020-19197-8

 4. The author collected the platelets, stained them and reconstituted the blood. This approach may not fully replicate physiological conditions and introduces additional complexity. A more representative method would involve staining platelet markers directly within whole blood to preserve native interactions and simplify the procedure.

 5. The timing of blood usage post-collection is an important factor. Platelet activity diminishes over time, which could impact thrombus formation outcomes. It would be helpful if the authors could clarify the timeframe within which the blood was used after collection. Supporting literature has demonstrated significantly reduced thrombus formation efficacy beyond 2 hours, even in identical setups: Akther F et al [2022(Ref-5)] https://advanced.onlinelibrary.wiley.com/doi/full/10.1002/adbi.202101316

 6. The authors propose that the device could be applied to embolism modeling and drug testing. To validate this application, it is recommended that at least one antiplatelet or antithrombotic drug be tested to demonstrate the model's responsiveness and reliability in a pharmacological context.

 Minor Corrections:

1. Terms such as in vivo should be consistently italicized throughout the manuscript. Please review and revise accordingly.
2. Ambiguity in Language (Introduction, Page 3, Line 5): The sentence beginning “However, when these…” is unclear, particularly the phrase “clotting occurs inappropriately inside undamaged blood vessels.” Please rephrase for clarity and accuracy.
3. Terminology Correction (Page 4, Line 1): The sentence starting with “Acute cardiovascular events…” may more accurately refer to chronic cardiovascular events. Please reassess and revise this statement if necessary.

Overall, the paper presents a promising direction in microfluidic thrombosis modelling. With further clarification, validation, and refinement, it could significantly contribute to the field. I look forward to seeing the revised version.

Is the rationale for developing the new method (or application) clearly explained?

Yes

Is the description of the method technically sound?

No

Are the conclusions about the method and its performance adequately supported by the findings presented in the article?

No

If any results are presented, are all the source data underlying the results available to ensure full reproducibility?

Yes

Are sufficient details provided to allow replication of the method development and its use by others?

Yes

Reviewer Expertise:

microfluidic, lab-on-chip, cardiovascular, flaviviruses, vaccine development

I confirm that I have read this submission and believe that I have an appropriate level of expertise to confirm that it is of an acceptable scientific standard, however I have significant reservations, as outlined above.

## References

[1] : An in vitro model of foam cell formation induced by a stretchable microfluidic device. *Sci Rep* .2019;9(1) : 10.1038/s41598-019-43902-3 7461 10.1038/s41598-019-43902-3 31097769 PMC6522483

[2] : An intimal-lumen model in a microfluidic device: potential platform for atherosclerosis-related studies. *Lab Chip* .2025;25(3) : 10.1039/d4lc00868e 354-369 10.1039/d4lc00868e 39698809

[3] : A novel human arterial wall-on-a-chip to study endothelial inflammation and vascular smooth muscle cell migration in early atherosclerosis. *Lab Chip* .2021;21(12) : 10.1039/d1lc00131k 2359-2371 10.1039/d1lc00131k 33978037

[4] : Modeling early stage atherosclerosis in a primary human vascular microphysiological system. *Nat Commun* .2020;11(1) : 10.1038/s41467-020-19197-8 5426 10.1038/s41467-020-19197-8 33110060 PMC7591486

[5] : Atherothrombosis-on-Chip: A Site-Specific Microfluidic Model for Thrombus Formation and Drug Discovery. *Adv Biol (Weinh)* .2022;6(7) : 10.1002/adbi.202101316 e2101316 10.1002/adbi.202101316 35666057

# Reviewer report: Reviewer response for version 1

**DOI:** 10.5256/f1000research.174562.r369848

**Published:** 2025-04-04

**Authors:** Kaushik N. Shankar

**Affiliations:** 1 University of Pennsylvania, Philadelphia, Pennsylvania, USA

This study presents a thrombosis-on-a-chip model that has the potential to improve the predictive power of preclinical anti-thrombotic drug testing while reducing reliance on animal models. By using TEAC and microfluidics, the authors introduce a system that is directly applicable to human blood.

 The work aligns well with ongoing efforts to develop and enhance individual specific drug development for thrombotic disorders. The inclusion of a step-by-step methodological guide and video tutorials is particularly valuable, as it enhances accessibility and reproducibility for other researchers looking to adopt this model.

 I have a few suggestions, if addressed, could further strengthen the study's claims and practical utility.

 1. While the model replicates a shear stress that is within the physiological range for arteries, physiological blood flow is highly dynamic, with pulsatile shear stress and varying flow patterns. Some discussion on how the model captures these complexities, or whether any adjustments are needed for specific applications, would provide additional clarity on its physiological relevance.

 2. It would be useful to discuss how stable the TEAC remains over extended experimental durations. Does the construct maintain consistent mechanical and biological properties over time? For example, even within a short timeframe, mechanical properties of the TEAC could change upon exposure to blood flow/ pressure.

 3. The authors state that this model provides a more reliable and reproducible thrombotic/hemostatic response in comparison to in vivo animal models. Showing how the model actually responds to known antithrombotic therapies and comparing those results to existing in vivo data, even for a single case, would add significant value as a proof of concept. If the model is to be used in a practical setting to provide improved translational outcomes, this comparison would make the case much stronger, though it is understandable if this falls within the scope of future work.

Is the rationale for developing the new method (or application) clearly explained?

Yes

Is the description of the method technically sound?

Yes

Are the conclusions about the method and its performance adequately supported by the findings presented in the article?

Partly

If any results are presented, are all the source data underlying the results available to ensure full reproducibility?

Yes

Are sufficient details provided to allow replication of the method development and its use by others?

Yes

Reviewer Expertise:

multiscale modeling, thrombosis, microfluidics

I confirm that I have read this submission and believe that I have an appropriate level of expertise to confirm that it is of an acceptable scientific standard, however I have significant reservations, as outlined above.

# Reviewer report: Reviewer response for version 1

**DOI:** 10.5256/f1000research.174562.r369852

**Published:** 2025-03-31

**Authors:** Navaneeth Krishna Rajeeva Pandian

**Affiliations:** 1 Harvard Medical School, Wyss Institute at Harvard, Boston, USA

The authors have submitted a methods manuscript on a humanized thrombus on a chip that would help reduce and replace animal models. This is an excellent paper, considering the increased use of microfluidic organ-chip platforms, especially vessel-on-chip platforms, to study thrombosis. This paper would help create reliable and reproducible results from different labs, a challenge faced in the field.

 There are some clarifications needed before this paper is accepted for indexing:

1. The authors claim this model is a realistic arterial model. It consists of the endothelial lining of the intimal layer and the smooth muscle cell layer containing ECM proteins of the medial layer. To demonstrate the in vitro model and its operation, a stained confocal image of these cells and layers (if possible, a 3D view) would be helpful.
2. The staining protocol used in this paper is cumbersome as there are straightforward protocols to stain for platelets (CD41) in whole blood. CD41 antibodies can be directly added to the whole blood to stain for platelets compared to the separation process followed by reconstitution used in this manuscript. It would be helpful if the authors can demonstrate the advantages of the current protocol to the existing protocols to stain for platelets in whole blood.
3. All images need labeling, making it easier for the readers to follow the figure panels. Figure captions also need to be consistent (for eg. Biorender is acknowledged at the beginning in some and at the end in some).

Is the rationale for developing the new method (or application) clearly explained?

Yes

Is the description of the method technically sound?

Partly

Are the conclusions about the method and its performance adequately supported by the findings presented in the article?

Yes

If any results are presented, are all the source data underlying the results available to ensure full reproducibility?

Yes

Are sufficient details provided to allow replication of the method development and its use by others?

Yes

Reviewer Expertise:

Coagulation, microfluidics, organ-chips, endothelial biology

I confirm that I have read this submission and believe that I have an appropriate level of expertise to confirm that it is of an acceptable scientific standard, however I have significant reservations, as outlined above.

# Reviewer report: Reviewer response for version 1

**DOI:** 10.5256/f1000research.174562.r369847

**Published:** 2025-03-21

**Authors:** Michael Emerson

**Affiliations:** 1 Imperial College London, London, UK

This article very clearly describes a novel humanised thrombus-on-a-chip model involving a 3D printed microfluidic flow chamber which incorporates endothelial cells and fluorescently labelled human blood. Critically there is detail of how to standardise injury which leads to variability in comparable mouse injury models. The model has the potential to identify novel therapeutic drugs through improved pre-clinical testing and the potential to incorporate diverse blood donors - this is critical due to the lack of inclusion of women and minoritised communities from clinical research and their exclusion almost entirely from many pre-clinical assays. I had a few comments that could be considered as discussion points:

 It would be useful to validate the model under blinded conditions with drugs of proven (and critically disproven) clinical efficacy.

 Currently the model is described as a tool for drug testing which would be limited to active drugs i.e. not pro-drugs that require metabolic conversion in vivo

 It would be useful to discuss the implications of "patient" modelling in addition to the more general "humanisation". There is scope for working with patients with defined CVD and those at higher risk of CVD, This could include patients on therapy to create more accurate mimicking of drug exposure and the use of patient-derived cells such as endothelial colony forming cells to create a fully relevant patient model. The use of human blood and cell lines only takes us half way there and continued development towards a full patient model will be yet another step forward.

Is the rationale for developing the new method (or application) clearly explained?

Yes

Is the description of the method technically sound?

Yes

Are the conclusions about the method and its performance adequately supported by the findings presented in the article?

Yes

If any results are presented, are all the source data underlying the results available to ensure full reproducibility?

Yes

Are sufficient details provided to allow replication of the method development and its use by others?

Yes

Reviewer Expertise:

Cardiovascular pharmacology, 3Rs science and platelet biology

I confirm that I have read this submission and believe that I have an appropriate level of expertise to confirm that it is of an acceptable scientific standard.
